# Mitigation of hepatic and gastric impairments induced by flunixin meglumine through co-administration with alpha lipoic acid in male rats

**DOI:** 10.1186/s12917-025-04751-7

**Published:** 2025-05-28

**Authors:** Zeynab Kh. El-Maddawy, Abdel-wahed A. Mashalla, Sulaiman Mohammed Alnasser, Abd El-Salam F. El-Sawy, Walied Abdo, Maher A. Kamel, Meshal Alotaibi, Mohsen A. Khormi, Ali M. Aborasain, Hanan H. Abd-El-Hafeez, Amal A. Awad

**Affiliations:** 1 Veterinary Pharmacology Department, Faculty of Veterinary Medicine, Alexandria University, Alexandria, Egypt; 2 Toxicology and Forensic Medicine Department, Faculty of Veterinary Medicine, Omar Al-Moukhtar University, El-Bedia, Libya; 3 Department of Pharmacology and Toxicology, College of Pharmacy, Qassim University, 51452 Qassim, Saudi Arabia; 4 Department of Pathology -Faculty of Veterinary Medicine, Kafr El-Sheikh University, Kafr El-Sheikh, Egypt; 5 Department of Biochemistry, Medical Research Institute, Alexandria University, Alexandria, Egypt; 6 Research Projects unit, Pharos University in Alexandria, Alexandria, 21648 Egypt; 7 Department of Pharmacy Practice, College of Pharmacy, University of Hafr Albatin, Hafer Al Batin, 39524 Saudi Arabia; 8 Department of Biology, College of Science, Kingdom of Saudi Arabia, Jazan University, P.O. Box. 114, Jazan, 45142 Saudi Arabia; 9 Department of Cell and Tissues, Faculty of Veterinary Medicine, Assiut University, Assiut, 71526 Egypt

**Keywords:** Flunixin meglumine, Alpha lipoic acid, Antioxidant status, Pro-inflammatory cytokines, Rats

## Abstract

Long term use of Flunixin meglumine produces many gastric and hepatic hazards. The current study aimed to investigate using Alpha lipoic acid (ALA) for treating flunixin meglumine (FM)-induced liver and gastrointestinal problems in male rats. FM alternated with ALA for 14 and 56 days in the experiment. This study divided 72 male rats into six groups, 12 rats for each group. Group 1 (control) received saline and distilled water, Group 2 (ALA) received alpha lipoic acid orally at 100 mg/kg bwt, Group 3 (FM-2.5) received Flunixin meglumine subcutaneously at 2.5 mg/kg bwt, Group 4 (FM-5) received Flunixin meglumine subcutaneously, Group 5 (FM-2.5 and ALA) received FM and ALA, and Group 6 received FM and ALA. Elevated white blood cell (WBC) concentrations, ALT, AST, ALP, pro-inflammatory cytokines (NF-κB, TNF-α, HMG), malonaldehyde (MDA), and significant reductions in hepatic and gastric total antioxidant capacity (TAC) were observed. At weeks 4 and 8, FM-5-treated groups had a lower stomach index weight. These changes improved when Groups 5 and 6 used ALA and FM. ALA treatment reduced WBCs, ALT, AST, ALP, NF-κB, TNF-α, HMG, MDA, TAC, and stomach index weight gains in FM-5-treated groups. Finally, biochemical markers and stomach index volume showed liver and stomach dysfunctions in male rats after FM injections. The simultaneous administration of ALA greatly reduced these deficits, suggesting it may prevent FM-related hepatic and gastrointestinal diseases.

## Introduction

Pain can be defined as an unpleasant sensory and emotional sensation associated with actual or possible damage to bodily tissues. Animals undergoing treatments or illness models are likely to have a response that has the potential to induce pain in humans [1]. Non-steroidal anti-inflammatory medications (NSAIDs) have traditionally been relied upon for their modest pain-relieving and anti-inflammatory properties rather than for the treatment of persistent pain. Nevertheless, recent generations of NSAIDs exhibit significant analgesic qualities that coincide with the pain-relieving effects of opioids. The benchmark for comparison in this medicine category is aspirin, as established by Flecknell et al. and Harder and An1 [2, 3]. Nonsteroidal anti-inflammatory drugs (NSAIDs) exert their effects by the inhibition of the inflammatory response and the suppression of kinin and prostaglandin synthesis. These medications exhibit different degrees of effectiveness such as antipyretics, analgesics, and anti-inflammatory medicines. Novel nonsteroidal anti-inflammatory drugs (NSAIDs) such as ketoprofen, carprofen, ketorolac, flunixin meglumine, and meloxicam have demonstrated efficacy in relieving acute pain, especially pain caused by surgical operations [4].

The adverse effects linked to NSAIDs include ulcers of the gastrointestinal tract, impairment of platelet aggregation, nephrotoxicity, protracted labor, fetal abnormalities, blood problems, compromised bone repair, and hepatotoxicity. These adverse effects are commonly seen with prolonged usage and are seldom documented with short-term dosing [2]. Inger et al. [5] reported that its mode of action is the inhibition of cyclooxygenase, an enzyme that is responsible for the synthesis of prostaglandins from arachidonic acid.

Flunixin, a derivative of reduced nicotinic acid, is a nonsteroidal anti-inflammatory medication (NSAID) widely used in veterinary medicine since the late 1970 s. The primary mechanism of action of flunixin meglumine (FM) is the inhibition of prostaglandin synthesis, resulting in strong anti-inflammatory, analgesic, and antipyretic properties. Flunixin meglumine (FM) is extensively used in the management of certain animal disorders such as fever, mastitis, endotoxemia, and lameness [6]. The veterinary medication flunixin meglumine is classified as a non-steroidal anti-inflammatory medicine (NSAID). With its analgesic and antipyretic characteristics, this product is especially designed for application in horses, cattle, and swine. Only a licensed veterinarian is authorized to prescribe this medicine. The drug flunixin meglumine is frequently used to treat inflammation and discomfort caused by musculoskeletal diseases in horses, as well as to alleviate visceral pain linked to colic [7]. Flunixin meglumine inhibits both cyclooxygenase- 1 (COX- 1) and COX- 2 enzymes without discrimination. Approval has been granted for the therapeutic management of many inflammatory and non-inflammatory disorders in both human and animal subjects, encompassing arthritis, post-operative pain, and post-traumatic pain [8]. Arachidonic acid is converted into prostanoids, including prostaglandins (PGs), prostacyclins, and thromboxane, by the enzyme cyclooxygenase (COX). Prostanoids are essential mediators that control many biological processes in the gastrointestinal, cardiovascular, urogenital, and neurological systems. Moreover, they carry out a crucial function in immunological responses and the process of inflammation [9]. The medication flunixin is quite potent, exhibiting robust anti-inflammatory and analgesic characteristics [10]. This compound demonstrates a predilection for suppressing cyclooxygenase- 1 and is mostly prescribed for the treatment of acute and surgical pain. Adverse effects including acute renal failure, increased serum alanine aminotransferase activity, and the formation of gastro-duodenal ulcers have been reported in dogs treated with flunixin [10–12]. The findings of Fujiwara et al. Fujiwara, et al. [13] and Wolz and Krieglstein Wolz and Krieglstein [14], indicate that ALA, commonly known as lipoate, thioctic acid, or 6,8-dithiooctanoic acid, is a chemical molecule composed of eight carbon atoms and two sulfur atoms. Large-chain lipoic acid (ALA) has attracted considerable attention as a preventive agent against many clinical disorders caused by oxidative stress [15, 16]. ALA's antioxidative properties are attributed to its ability to efficiently remove reactive oxygen species, bind metal ions, replenish natural antioxidants, reduce lipid oxidation, and repair damaged biomolecules caused by oxidation [17, 18].

The literature states that ALA has solubility in both water and fat, rendering it very efficient in the reduction of free radicals, including lipid peroxides, in both hydrophilic and lipophilic conditions. Saad, et al. [19] and Gorąca, et al. [20], have reported that ALA exhibits the distinctive characteristic of solubility in both water and fat substrates. ALA's great efficiency in fighting free radicals, especially lipid peroxides, in different cellular settings, including both hydrophilic and lipophilic domains, is attributed to this feature. The study found that ALA is a versatile antioxidant that inhibits the generation of free radicals, restores insulin sensitivity, regenerates other antioxidants such as vitamins E and C, enhances intracellular glutathione levels, and prevents lipid peroxidation. However, it was also determined that both ALA and dihydrolipoic acid can function as powerful antioxidants by scavenging several free radicals in both the hydrophilic and lipophilic phases of the cell. The research conducted by Packer and Cadenas [21], indicates that ALA possesses several advantageous antioxidant characteristics. The compound efficiently suppresses the liberation of free radicals, reinstates the responsiveness of insulin, regenerates other antioxidants such as vitamins E and C, increases the amounts of glutathione inside cells, and serves to prevent lipid peroxidation. In addition, the research carried out by Yi et al. Yi, et al. [22] demonstrated that both ALA and dihydrolipoic acid exhibit strong antioxidant properties by neutralizing different free radicals in both water-soluble and fat-soluble cellular settings. Kan et al. Kan, et al. [23] suggested that ALA operates as an inhibitor of oxidative stress by neutralizing reactive oxygen species (ROS), restoring natural antioxidants, healing oxidative damage, and binding to metal ions.

The aim of this study was to investigate the effects of flunixin meglumine, either as a standalone treatment or in conjunction with alpha lipoic acid as a protective agent, on the levels of liver enzymes, hepatic and stomach oxidative status, pro-inflammatory cytokine levels, and tumor pathological alterations.

## Material and methods

### Ethical approval

The study protocol was approved by the local ethical committee on Animal use from the Faculty of Veterinary Medicine at Alexandria University. All animal procedures were performed in accordance with the Guidelines for Care and Use of Laboratory Animals of ICLAS- 2015. All procedures and experiments were accepted by the local ethics committee of animal use from the Faculty of Veterinary Medicine, Alexandria University-Institutional Animal Care and Use Committee (AU-IACUC) under approval number (5/2023/241). All methods were performed in accordance with the relevant guidelines and regulations and the study is reported in accordance with ARRIVE guidelines [24].

### Animals and experimental design

Male rats (Rattus norvegicus), weighing an average of 180 ± 20 g, and aged around 150 days, were acquired from the Medical Research Institute at Alexandria University, Egypt, for use in this investigation. In controlled environmental settings, the rats were individually housed in plastic cages maintained at a temperature of 23 ± 2 °C, relative humidity of 55%, and exposed to a 12-h light/dark cycle. Ad-libitum provision of standard laboratory food and drink was maintained throughout the trial. Before the trial began, a two-week acclimatization phase was undertaken, during which the rats were maintained under constant sanitary and environmental settings [25, 26].

The criteria were consistently upheld for the whole duration of the experiment. The rats were partitioned into six-matched groups, with 12 rats in each group, in the following manner: Among the experimental groups, Group 1 (control) was administered saline subcutaneously at a dosage of 2 ml/kg body weight daily for 14 consecutive days. Additionally, they were given distilled water orally by stomach tube once daily for 56 consecutive days, which served as a carrier for alpha lipoic acid (ALA). In Group 2, ALA was administered orally at a dosage of 100 mg/kg body weight once daily through a stomach tube for 56 consecutive days. Simultaneously, saline was subcutaneously administered daily at a dosage of 2 ml/kg body weight for 14 consecutive days. Group 3 (FM- 2.5) rats received a subcutaneous injection of Flunixin meglumine (obtained from Shering-Plough Animal Health Co, USA) at a dosage of 2.5 mg/kg body weight once daily for 14 consecutive days, following the recommended regime by by Erpek et al. and Tubbs et al. Tubbs, et al. [27], Erpek, et al. [28]. They also received oral administration of saline (2 ml/kg body weight) once daily for 56 consecutive days. Group 4 (FM- 5) rats received a subcutaneous injection of Flunixin meglumine at a dosage of 5 mg/kg body weight once daily for 14 consecutive days, along with oral supplementation of saline (2 ml/kg body weight) once daily for 56 consecutive days [29] Rats in Group 5 (FM- 2.5 and ALA) received Flunixin meglumine twice daily at a dosage of 2.5 mg/kg body weight through subcutaneous injection for 14 consecutive days. Concurrently, patients were orally administered alpha lipoic acid (ALA) at a dosage of 100 mg/kg body weight through a stomach tube once daily for 56 consecutive days. Lastly, rats in Group 6 (FM- 5 and ALA) administered subcutaneous injections of Flunixin meglumine at a dosage of 5 mg/kg body weight once daily for a period of 14 consecutive days. Concurrently, the participants received alpha lipoic acid (ALA) orally at a dosage of 100 mg/kg body weight delivered through a stomach tube once daily for 56 consecutive days.

### Collection of blood

Each rat was weighed and euthanized after 4 and 8 weeks of the experiment. Serum separation was achieved by collecting blood samples and subjecting them to direct centrifugation at 1006 xg/10 min. Using heparinized micro-hematocrit tubes under ketamine/xylazine anesthesia (7.5 and 1.0 mg/kg intraperitoneal infusion)., blood samples were obtained from the retro-orbital vein of the rat's eye before the rodent was euthanized by cervical dislocation. Blood samples were obtained from each animal without anticoagulant in sterile test tubes. The samples were allowed to coagulate at room temperature for 2 h in a slope posture and then centrifuged at 3000 revolutions per minute for 15 min to separate the serum. Aqueous serum samples were kept at − 20 degrees Celsius until they were used for biochemical analysis.

### Tissue sampling

Following a macroscopic examination, the livers and stomachs of each rat were separately removed and weighed. The index weight of the dissected organs was determined using the formula I.W. = [organ weight (gm) × body weight (gm)] × 100 [30].

Subsequently, the livers and stomachs of the `` from each group were divided into two portions. One portion was rapidly frozen using liquid nitrogen and kept at a temperature of − 80 ◦C for the purpose of biochemical analysis of oxidative stress and pro-inflammatory markers. The other portion was preserved in a 10% formalin for histopathological investigations and immunohistochemical assessment of NF-κB p65 and Caspase- 3. Once the macroscopic inspection was completed, the livers and stomachs of each rat were meticulously dissected and weighed. The organ index weight was determined by calculating the ratio of organ weight (gm) to body weight (gm) and then multiplying the result by 100, following the approach outlined by Matousek [30].

### Determination of pro-inflammatory cytokines examination

Hepatic and stomach inflammation were evaluated by measuring the concentrations of Nuclear Factor kappa B (NFĸB), Tumor Necrosis Factor alpha (TNF-α), and Rat High Mobility Group Protein B1 (HMG) in tissue lysates. ELISA kits of rat-specific origin from Chongqing Biospes Co., Ltd (catalogue numbers BYEK304, BEK1214, and BYEK2511, respectively) were used to quantify these markers following the manufacturer's instructions.

#### Determination of total protein

An adapted version of the technique described by Lowry OH et al. [31] was employed to quantify the protein content in the liver and stomach. The observed colour is assumed to result from the formation of a compound between the alkaline copper phenol reagent and the tyrosine and tryptophan residues present in the protein within the sample. Each sample's protein concentration was determined by comparing it to a standard curve generated using bovine serum albumin, following the manufacturer's methods.

### Calculation

$$\text{The total protein concentration }\left(\text{mg}/\text{ml}\right)= \frac{\text{The total protein amount }\left(\text{mg}\right)}{\text{The sample volume }\left(\text{ml}\right)}$$

#### Determination of nuclear factor ĸb

This kit was developed using conventional sandwich enzyme-linked immunosorbent assay method. The 96-well plates were covalently coated with the purified anti-NFεb-p65 antibody. Based on the methodology developed by Chen, et al. [32, 33].

#### Determination of rat TNF

These kits were developed using sandwich enzyme-linked immunosorbent assay technique. Polyclonal antibody targeting TNFα was used as a pre-coat on 96-well plates. Biotin-conjugated anti-TNFα polyclonal antibody was employed as the detecting antibody. In accordance with the methodology acquired by Nolan, et al. [33–36].

#### Determination of rat high mobility group proteinB1 (HMG)

The 96-well plates were covalently coated with the purified anti-HMGB1 antibody. The HRP-conjugated anti-HMGB1 antibody was employed as the detecting antibody. In accordance with the methodology established by Ferrari, et al. [37–40].

### Gastric juice contents assessment

To eliminate any solid debris, the stomach juice obtained from each animal was subjected to centrifugation at 3000 revolutions per minute for 10 min. The volume of the resulting supernatant was then measured.

#### Assessment of pepsin activity

Hemoglobin was used to measure pepsin level following the procedure developed by Anson and Mirsky Anson and Mirsky [41]. The transparent filtrate was isolated and measured at an absorbance of 650 nm. Pepsin content levels were reported as micromoles of tyrosine released as μM of tyrosine liberated/ml.

#### Assessment of mucin

Mucin levels were determined using the Corne Corne [42] technique with 0.1% alcian blue, prepared as a 0.16 M sucrose buffered with 0.05 M sodium acetate.

Dilute the dye and mucin combination by immersing it in 10 ml of a 0.5 M magnesium chloride solution for 2 h. Blue solution was quantified at a wavelength of 605 nm. The mucin concentration of the sample was quantified as the percentage of mg of sucrose.

### Biochemical analysis

Blood samples were obtained in plain tubes and then underwent centrifuged at a velocity of 10,000 revolutions per minute (r/min) for a period of 10 min. For further investigation of serum alanine aminotransferase (ALT), aspartate transaminase (AST), ALP, total protein (TP), and albumin content, the resultant serum was meticulously preserved at a temperature of − 20 °C. The measurements were performed according to the instructions given by the manufacturer of the corresponding analytic kits.

### Oxidative stress and antioxidants assays

Liver and stomach tissues were evaluated for oxidative stress and antioxidant activity by quantifying malondialdehyde (MDA) concentrations and total antioxidant capacity (TAC). Subsequently, the tissue samples were homogenized in cooled Tris–HCl buffer (pH 7.4) and centrifuged at 12,000 × g for 30 min at 4 °C. Analysis of the supernatant was conducted using spectrophotometry with diagnostic kits acquired from Biodiagnostic Co., Egypt. Lipid peroxidation (MDA) content in hepatic and gastric tissues was quantified by subjecting the samples to thiobarbituric acid in an acidic environment of reaction. The thiobarbituric acid-reactive product was formed by conducting the reaction at a temperature of 95 °C for a duration of 30 min. At a wavelength of 532 nm, the absorbance of the resultant pink colored light was measured. The quantification of lipid peroxidation was performed by measuring the nmol MDA/gm wet tissue using the extension coefficient of MDA (32.54) of the method outlined by OhkawaOhkawa, et al. [43]. The spectrophotometric measurement of total antioxidant capacity in the hepatic and gastric tissues was conducted using the technique defined by Koracevic, et al. [44]. For this aim, kits acquired from Biodiagnostic Chemical Co., Egypt were used. The procedure entailed the interaction of antioxidants present in the tissue samples with a predetermined quantity of hydrogen peroxide (H2O2) provided outside. A particular amount of the supplied hydrogen peroxide was removed by the antioxidants present in the samples. Colourimetric determination of the residual H2O2 was achieved by an enzymatic reaction that transformed 3,5-dichloro- 2-hydroxybenzensulphonate into a pigmented product.

### Gastric mucosal lesion determination

After painstakingly euthanizing each rat, the stomach was gently removed, and opened by a small incision along its greater curvature. Using an ice-cold saline solution, the stomachs were washed extensively to remove gastric contents and blood clots. Then, with the mucosal surface facing upward, the stomachs were delicately stretched on a glass window. Under light pressure, a second plate of glass was placed on top. A digital camera was used to take high-resolution pictures of the ulcer. Using Image J, a specialist image analysis program developed by the National Institutes of Health (NIH) in the USA, the ulcer area was quantified in square millimeters (mm [2]). Li, et al. [45] technique was used to measure the stomach lesion score.

### Histopathological examination

Rapid fixation in a 10% formalin solution was performed on small, fresh specimens obtained from the stomach and liver. Traditional paraffin-embedding procedures were used for handling the preserved specimens. Following the procedure outlined by Harries [46], paraffin blocks were sectioned into 5-micron thick pieces using a microtome and then stained with hematoxylin and eosin (H&E) for light microscopic analysis.

### Statistical analysis

S.P.S.S., 25 was used for statistical analysis of the collected data in a one-way analysis of variance. After finding a statistically significant difference, the means were compared using Tukey's test to assess intergroup variation. At (*P* < 0.05), the significant effect was established. Data were shown as the average plus or minus the standard error (SE) [47].

## Results

### Pro-inflammatory cytokines levels in liver and stomach

#### Nuclear factor kabba-B level

**Fig. 1:**
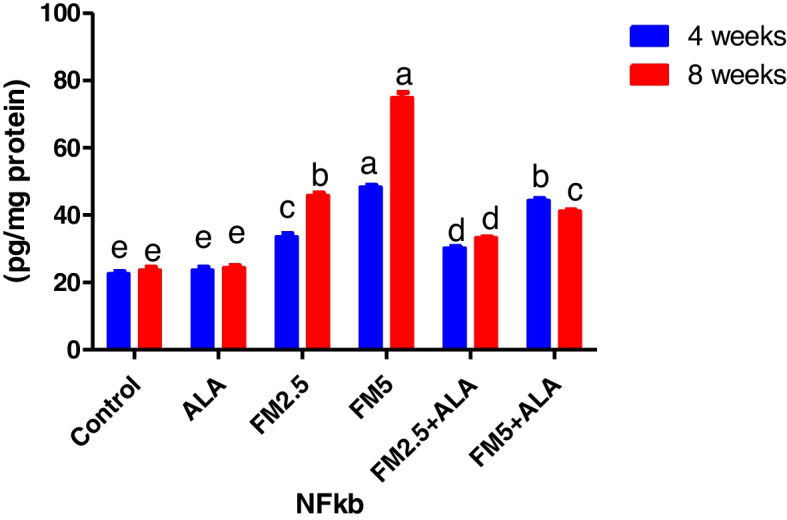
Effect of Flunixin meglumine (FM) and alpha lipoic acid (ALA) on hepatic NFk-b levels at the 4 th and 8 th weeks of the experiment. Values are expressed as the means ± S.E.M. (*n* = 6), showing a significant increase in hepatic NFk-b level in Flunixin meglumine-treated groups at dose levels of 2.5 and 5 mg/kg bwt in comparison to the control groups. In the FM- 5 + ALA and FM- 2.5 + ALA combination groups, the level was lower than in the treated groups alone

**Fig. 2:**
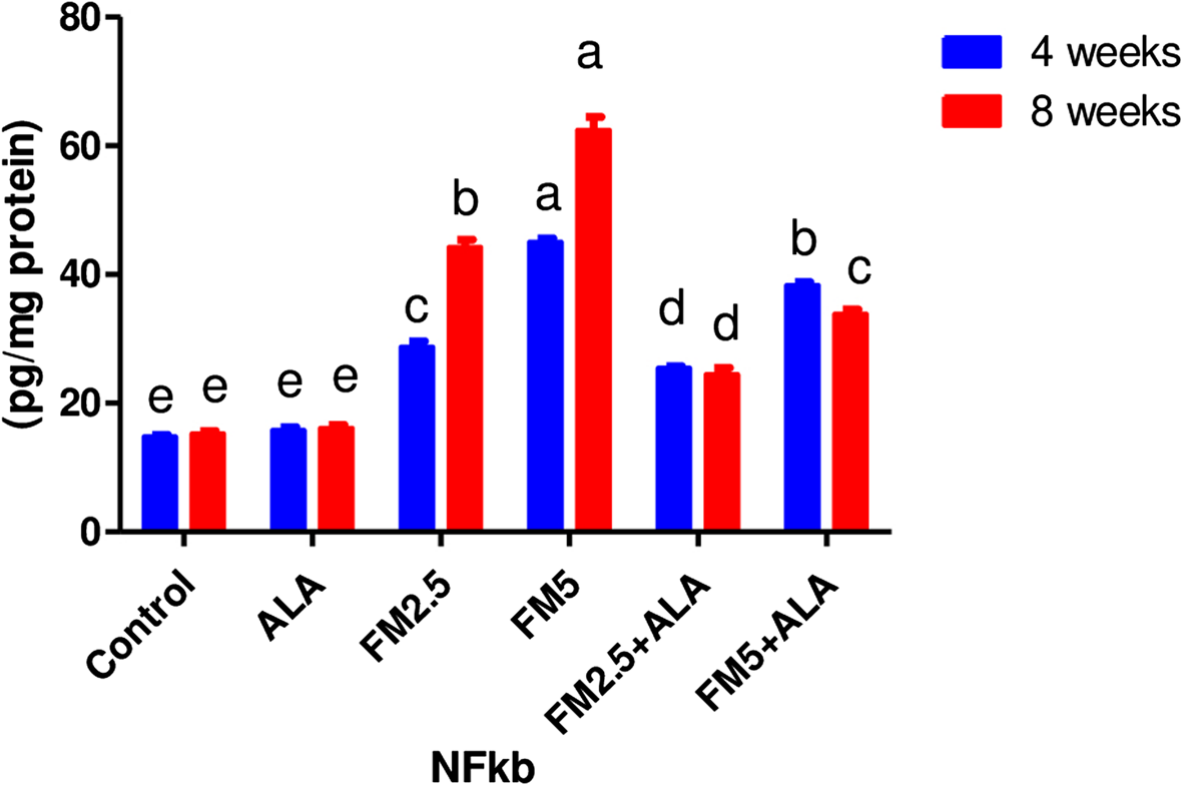
Effect of Flunixin meglumine (FM) and alpha lipoic acid (ALA) on gastric NFk-b levels in the 4th and 8th weeks of the experiment. The results are shown as the means ± S.E.M. for a group of six rats. The levels of NFk-b in the stomach were significantly higher in the groups that were given Flunixin meglumine at doses of 2.5 and 5 mg/kg bwt compared to the control groups. In the FM-5 + ALA and FM-2.5 + ALA combination groups, the level was lower than in the treated groups alone

Table 1 shows the level of nuclear factor kabba B (NFkb) in hepatic (Fig. 1) and gastric tissues (Fig. 2). During the 4 and 8 weeks of the experiment, flunixin meglumine significantly increased the NFkb in comparison to the control group at a dose level of 5 mg/kg bwt (double therapeutic dose). While the level of hepatic and gastric NFkb was lower in the FM- 5 + ALA combination group than it was in the FM5 treatment group by (8.22%, 44.23%) and (11.46%, 44.75%), respectively. Additionally, compared to the control group, the therapeutic dose of FM significantly raised NFkb levels, albeit not to the same extent as FM5. While the level of the hepatic NFkb was lower in the FM- 2.5 + ALA combination group than it was in the FM- 2.5 treatment group by (10%, 27%) and (14.88%, 45.77%), respectively.

**Table 1:** Effect of Flunixin meglumine (FM) and alpha lipoic acid (ALA) on the hepatic and gastric pro-inflammatory markers on 4^th^ and 8^th^ weeks of the experiment

Parameter	Period	Control	ALA	FM2.5	FM5	FM2.5 + ALA	FM5 + ALA
**Hepatic pro-inflammatory cytokines**							
NFkb	4 weeks	22.50 ± 0.73^e^	23.62 ± 0.88^e^	33.57 ± 0.91^c^	48.26 ± 0.64^a^	30.19 ± 0.44^d^	44.29 ± 0.56^b^
	8 weeks	23.73 ± 0.81^e^	24.36 ± 0.65^e^	45.76 ± 0.77^b^	74.98 ± 1.50^a^	33.29 ± 0.26^d^	41.11 ± 0.46^c^
TNF-α	4 weeks	9.61 ± 0.25^d^	9.95 ± 0.47^ cd^	14.76 ± 0.59^b^	21.68 ± 0.31^a^	11.60 ± 0.49^c^	14.90 ± 0.25^b^
	8 weeks	9.68 ± 0.27	10.64 ± 0.45^c^	16.18 ± 0.53^b^	24.62 ± 0.28^a^	15.02 ± 0.23^b^	15.62 ± 0.33^b^
HMGB1	4 weeks	135.67 ± 1.55^e^	136.50 ± 1.09^e^	217.85 ± 2.90^b^	320.01 ± 3.25^a^	177.4 ± 3.54^d^	200.28 ± 1.55^c^
	8 weeks	135.35 ± 2.74^e^	137.90 ± 2.80^e^	240.34 ± 3.09^b^	358.55 ± 1.73^a^	159.51 ± 1.64^d^	184.51 ± 4.01^c^
**Gastric pro-inflammatory cytokines**							
NFkb	4 weeks	14.75 ± 0.36^e^	15.74 ± 0.53^e^	28.71 ± 0.90^c^	45.02 ± 0.61^a^	25.42 ± 0.37^d^	38.32 ± 0.51^b^
	8 weeks	15.26 ± 0.41^e^	16.13 ± 0.53^e^	44.22 ± 1.18^b^	62.44 ± 2.09^a^	24.43 ± 1.08^d^	33.86 ± 0.68^c^
TNF-α	4 weeks	6.36 ± 0.24^c^	6.94 ± 0.25^c^	12.11 ± 0.57^b^	17.72 ± 0.32^a^	11.45 ± 0.26^b^	16.64 ± 0.34^a^
	8 weeks	6.84 ± 0.29^e^	6.93 ± 0.23^e^	18.85 ± 1.14^b^	27.79 ± 0.84^a^	11.87 ± 0.41^d^	15.44 ± 0.31^c^
HMGB1	4 weeks	91.24 ± 1.36^e^	90.31 ± 2.11^e^	190.63 ± 1.68^c^	265.79 ± 1.59^a^	170.78 ± 0.31^d^	254.31 ± 0.28^b^
	8 weeks	104.20 ± 0.61^f^	107.02 ± 1.21^e^	283.71 ± 0.20^b^	383.80 ± 0.49^a^	146.76 ± 0.36^d^	232.37 ± 0.19^c^

^*^Means carrying different letters with the same raw are significantly different (*p* ≤ 0.05)

#### Tumer necrosis factor level-alpha

**Fig. 3:**
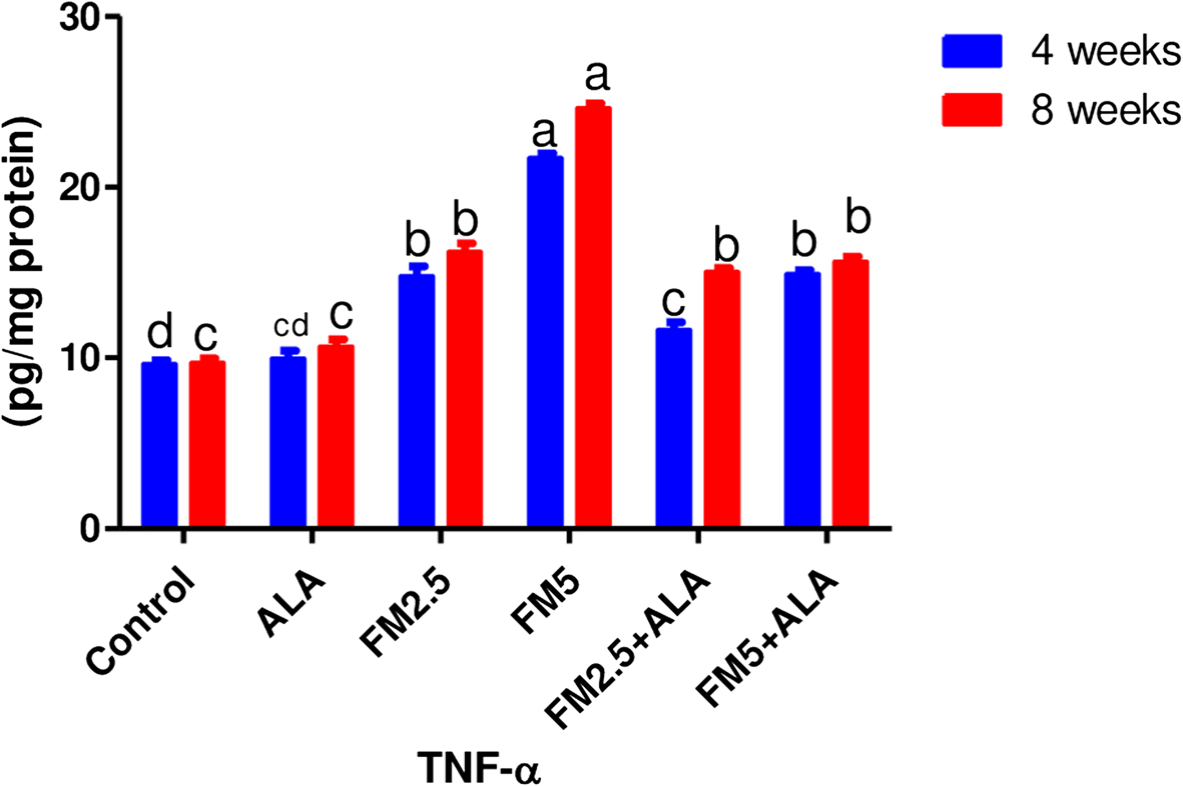
Effect of Flunixin meglumine (FM) and alpha lipoic acid (ALA) on hepatic TNF-α levels on the 4th and 8th weeks of the experiment. Values are expressed as means ± S.E.M. (*n* = 6), showing a significant increase in hepatic TNF-α level in Flunixin meglumine-treated groups at a dose level of 2.5 and 5 mg/kg bwt in comparison to the control groups. In the FM-5 + ALA and FM-2.5 + ALA combination groups, the level was lower than in the treated groups alone

Table 1 shows the level of TNF-α in hepatic tissues (Fig. 3). After 4 and 8 weeks, flunixin meglumine significantly raised TNF levels compared to the control group at a dose of 5 mg/kg bwt (twice the therapeutic dose). In contrast, the level was lower in the FM- 5 + ALA combination group than it was in the FM5 treatment group by 31.27% and 36.56%, respectively during the 4 th and 8 th weeks of the experiment, the administration of FM at 2.5 mg/kg bwt significantly increased TNF levels in comparison to the control group. Meanwhile, the level of the TNF-α in hepatic tissues was lower in the FM- 2.5 + ALA combination group than it was in the FM2.5 treatment group during the 4 th week by 21.41%.

**Fig. 4:**
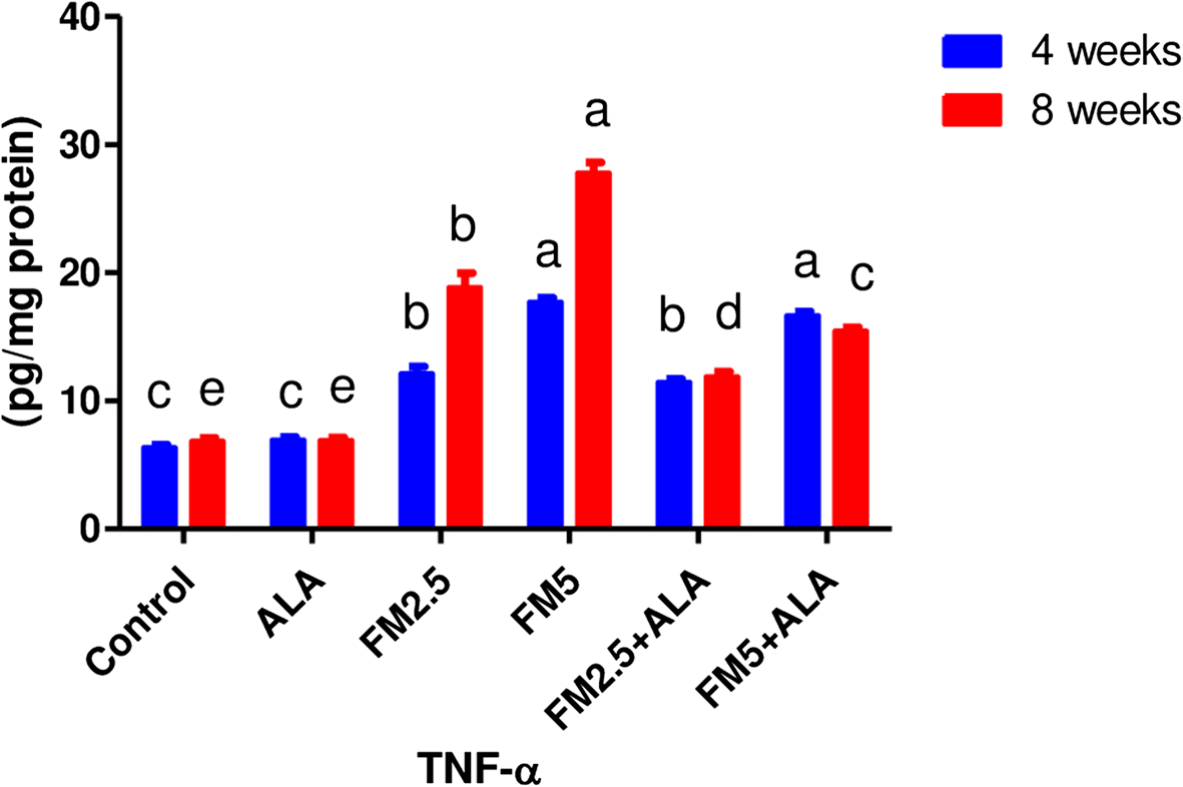
Effect of Flunixin meglumine (FM) and alpha lipoic acid (ALA) on gastric TNF-α levels on the 4th and 8th weeks of the experiment. Values are expressed as means ± S.E.M. (*n* = 6), showing a significant increase in gastric TNF-α level in Flunixin meglumine-treated groups at a dose level of 2.5 and 5 mg/kg bwt in comparison to the control groups. In the FM-5 + ALA and FM-2.5 + ALA combination groups, the level was lower than in the treated groups alone

The level of the TNF-α in gastric tissues (Fig. 4), Table 1 showed a significant increase in the TNF-α level in the FM2.5 and FM5 treated groups compared to the control of 175% and 306%, respectively. However, at the 8 th week, the levels decreased in the two combination groups with ALA by 37% and 44.4%, respectively.

#### High mobility group proteinB1 (HMG)

**Fig. 5:**
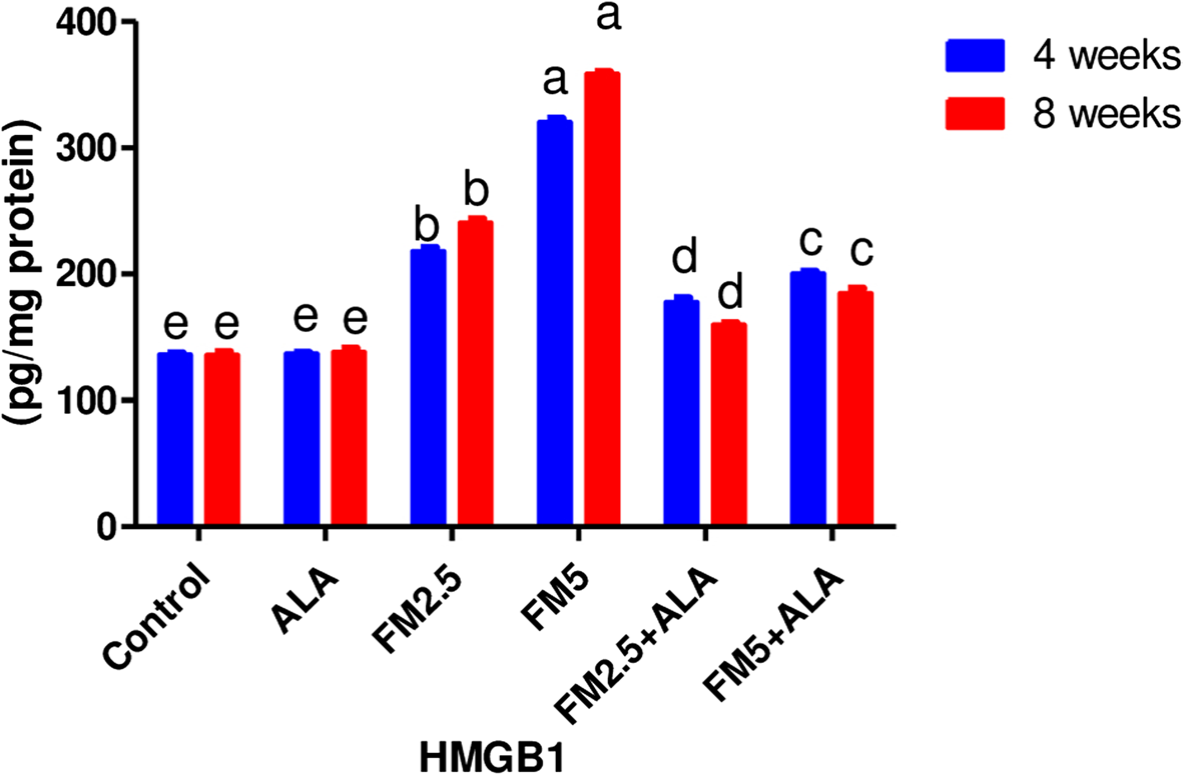
Effect of Flunixin meglumine (FM) and alpha lipoic acid (ALA) on hepatic HMGB1 levels at the 4th and 8th weeks of the experiment. Values are expressed as the means ± S.E.M. (*n* = 6), showing a significant increase in hepatic HMGB1 level in Flunixin meglumine-treated groups at dose levels of 2.5 and 5 mg/kg bwt compared to the control groups. In the FM-5 + ALA and FM-2.5 + ALA combination groups, the level was lower than in the treated groups alone

**Fig. 6:**
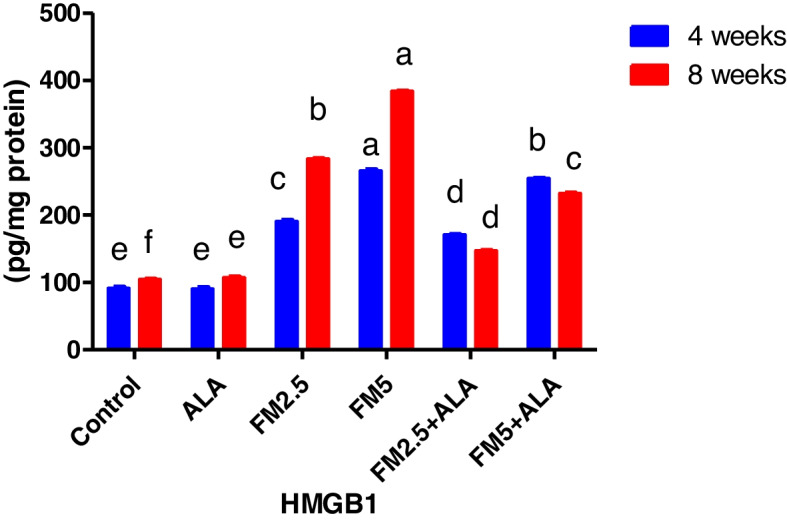
Effect of flunixin meglumine (FM) and alpha lipoic acid (ALA) on gastric HMGB1 levels in the 4th and 8th weeks of the experiment. The values shown are the means ± S.E.M. for a group of six rats. The results show that the levels of HMGB1 in the stomach were significantly higher in the groups that were given Flunixin meglumine at doses of 2.5 and 5 mg/kg bwt compared to the control groups. In FM-5 + ALA and FM-2.5 + ALA combination groups, the level was lower than in the treated groups alone

The findings reported in Table 1 and Figs. 5, 6 displayed the HMG (high mobility group protein B1) in the hepatic and gastric tissues. At 4 and 8 weeks into the experiment, the HMG level was much higher in the groups that were given flunixin meglumine at doses of 2.5 and 5 mg/kg bwt, which are the therapeutic and double therapeutic doses, respectively. In the meantime, the rise in HMG level at the twofold therapeutic dose level was greater than at the therapeutic level. The rise in HMG level at the twofold therapeutic dose level was greater than at the therapeutic dose level. Furthermore, this level significantly decreased in the two ALA combination groups by (18%, 33%) and (37%, 48%) in hepatic HMG and (10.4%, 4%) and (48.3%, 39%) in gastric HMG.

### Biochemical findings

**Fig. 7:**
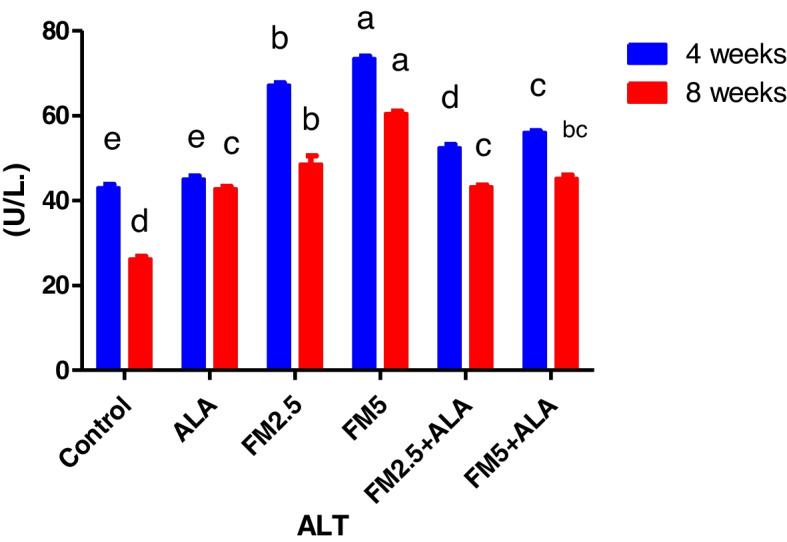
Effect of flunixin meglumine (FM) and alpha lipoic acid (ALA) on serum ALT levels in the 4 th and 8 th weeks of the experiment. The results are shown as the means ± S.E.M. for a group of six people. The serum ALT level was significantly higher in the groups that were given Flunixin meglumine at doses of 2.5 and 5 mg/kg bwt compared to the control groups. In the FM- 5 + ALA and FM- 2.5 + ALA combination groups, the level was lower than in the treated groups alone

**Fig. 8:**
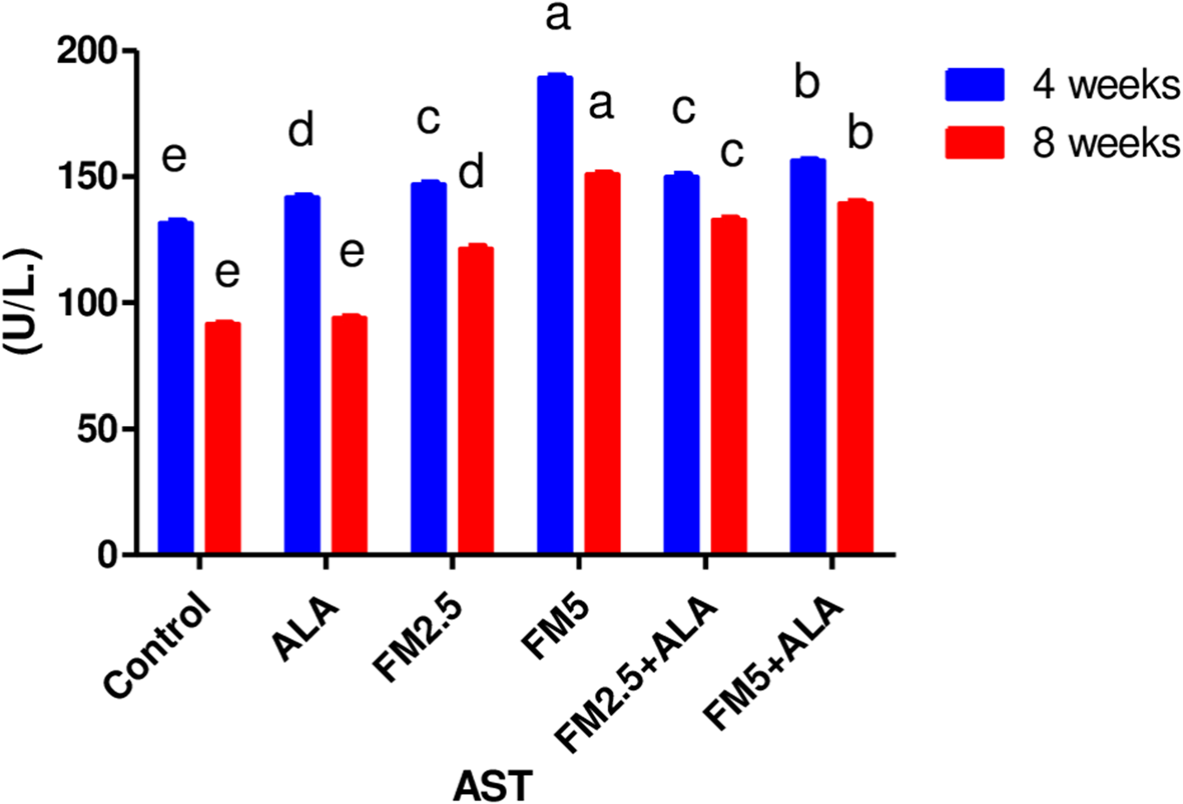
Effect of flunixin meglumine (FM) and alpha lipoic acid (ALA) on serum AST levels at the 4 th and 8 th weeks of the experiment. The results are shown as the means ± S.E.M. for a group of six rats. The serum AST level was significantly higher in the groups that were given Flunixin meglumine at doses of 2.5 and 5 mg/kg bwt compared to the control groups. In the FM- 5 + ALA and FM- 2.5 + ALA combination groups, the level was lower than in the treated groups alone

**Fig. 9:**
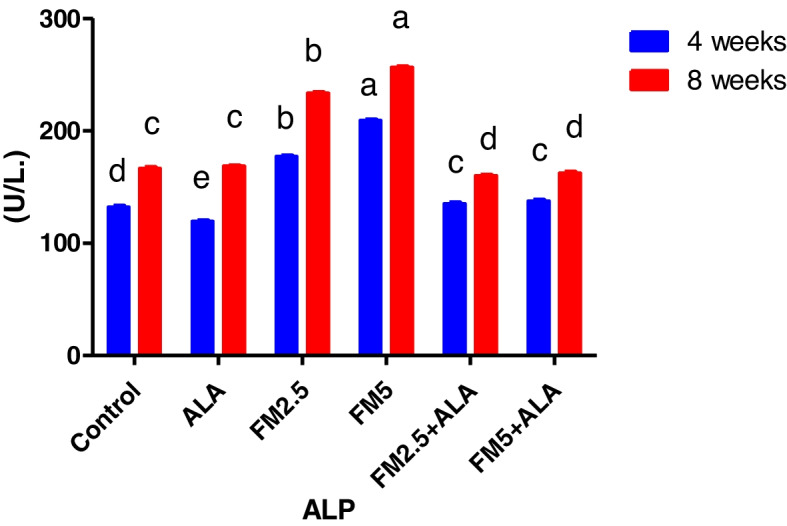
Effect of flunixin meglumine (FM) and alpha lipoic acid (ALA) on serum ALP levels at the 4 th and 8 th weeks of the experiment. The results are shown as the means ± S.E.M. for a group of six rats. The serum ALP level was significantly higher in the groups that were given Flunixin meglumine at doses of 2.5 and 5 mg/kg bwt compared to the control groups. In the FM- 5 + ALA and FM- 2.5 + ALA combination groups, the level was lower than in the treated groups alone

**Fig. 10:**
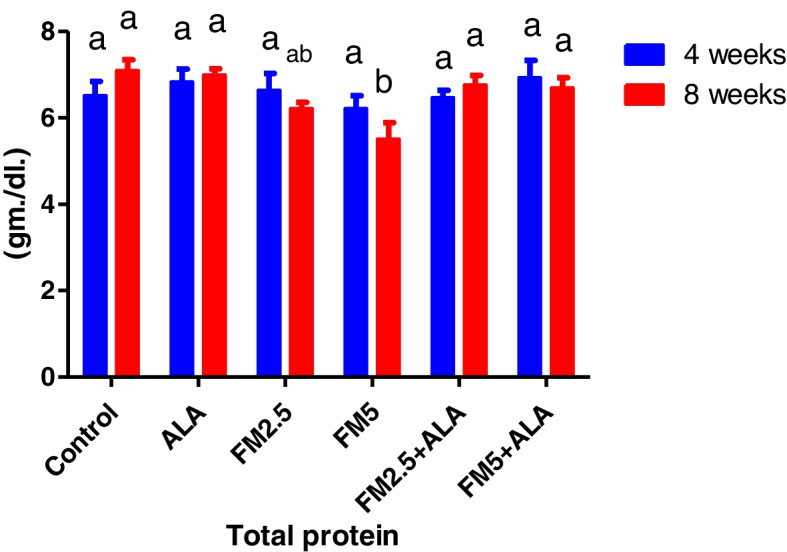
Effect of flunixin meglumine (FM) and alpha lipoic acid (ALA) on total protein level in the 4 th and 8 th weeks of the experimeFlunixin meglumine-treated groups, at dose levels of 2.5 and 5 mg/kg bwt, significantly reduced the total protein level compared to the control groups. ps. The levels were higher in the FM- 5 + ALA and FM- 2.5 + ALA combination groups than in the treated groups alone

**Fig. 11:**
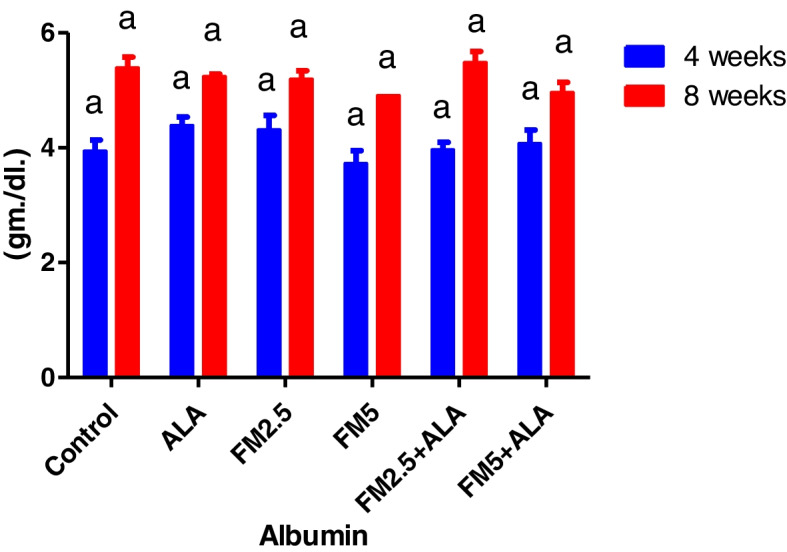
The experiment examined the impact of flunixin meglumine (FM) and alpha lipoic acid (ALA) on the albumin level during the 4 th and 8 th weeks. Values are expressed as the means ± S.E.M. (*n* = 6), showing non-significant change in the treated and control groups

**Fig. 12:**
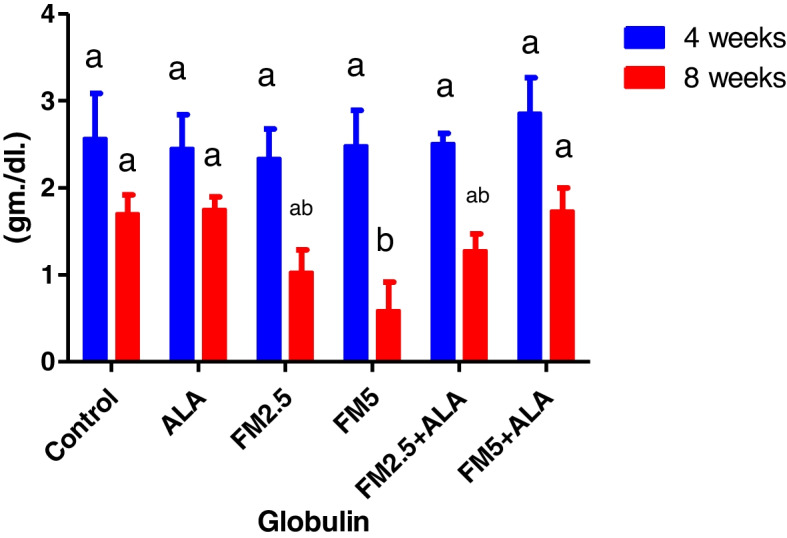
The impact of Flunixin meglumine (FM) and alpha lipoic acid (ALA) on the globulin level was observed during the 4 th and 8 th weeks of the experiment. There was a significant drop in globulin levels in the Flunixin meglumine-treated groups at dose levels of 2.5 and 5 mg/kg bwt compared to the control groups. The values are shown as the means ± S.E.M. (*n* = 6). The levels were higher in FM- 5 + ALA and FM- 2.5 + ALA combination groups than in the treated groups alone

The serum biochemical alterations were briefly recorded in Table 2. There were significant differences (*P* < 0.05) between the control group and groups 3 and 4, which were given FM at two different doses (2.5 and 5 mg/kg bwt), in the levels of ALT, AST, and ALP during the 4 th and 8 th weeks of the study. Additionally, the rise in enzyme levels was more pronounced in group 4 compared to group 3. In contrast, the liver enzyme levels of groups 5 and 6, which received FM in combination with ALA, decreased when compared to the groups that received FM alone. After 8 weeks of the study, the levels of total protein and globulin were significantly lower (*P* < 0.05) in the groups that were given FM compared to the control group. However, albumin concentration did not change during either experiment (Figs. 7, 8, 9, 10, 11 and 12).

**Table 2:** Effect of Flunixin meglumine (FM) and alpha lipoic acid (ALA) on the biochemical findings on 4^th^ and 8^th^ weeks of the experiment

Parameter	Period	Control	ALA	FM2.5	FM5	FM2.5 + ALA	FM5 + ALA
ALT	4 weeks	43.00 ± 0.81^e^	45.00 ± 0.82^e^	67.20 ± 0.54^b^	73.40 ± 0.65^a^	52.40 ± 0.81^d^	56.00 ± 0.47^c^
	8 weeks	26.20 ± 0.70^d^	42.80 ± 0.60^c^	48.60 ± 1.98^b^	60.50 ± 0.61^a^	43.20 ± 0.48^c^	45.20 ± 0.83^bc^
AST	4 weeks	131.40 ± 0.84^e^	141.63 ± 0.68^d^	146.75 ± 0.70^c^	189.00 ± 0.93^a^	149.80 ± 0.95^c^	156.25 ± 0.30^b^
	8 weeks	91.40 ± 0.35^e^	93.75 ± 0.54^e^	121.30 ± 0.87^d^	150.75 ± 0.54^a^	132.70 ± 0.73^c^	139.20 ± 0.70^b^
ALP	4 weeks	132.20 ± 0.70^d^	119.40 ± 0.42^e^	177.35 ± 0.63^b^	209.40 ± 0.42^a^	135.20 ± 0.56^c^	137.50 ± 0.75^c^
	8 weeks	166.60 ± 0.84^c^	168.50 ± 0.41^c^	233.60 ± 0.55^b^	256.50 ± 0.66^a^	160.00 ± 0.58^d^	162.48 ± 0.58^d^
Total protein	4 weeks	6.51 ± 0.33^a^	6.83 ± 0.30^a^	6.64 ± 0.39^a^	6.21 ± 0.30^a^	6.47 ± 0.17^a^	6.93 ± 0.40^a^
	8 weeks	7.09 ± 0.26^a^	6.99 ± 0.15^a^	6.22 ± 0.14^ab^	5.50 ± 0.39^b^	6.76 ± 0.22^a^	6.69 ± 0.24^a^
Albumin	4 weeks	3.94 ± 0.20^a^	4.39 ± 0.15^a^	4.31 ± 0.26^a^	3.72 ± 0.23^a^	3.96 ± 0.14^a^	4.07 ± 0.24^a^
	8 weeks	5.39 ± 0.19^a^	5.24 ± 0.05^a^	5.19 ± 0.15^a^	4.91 ± 0.00^a^	5.48 ± 0.20^a^	4.96 ± 0.18^a^
Globulin	4 weeks	2.57 ± 0.52^a^	2.45 ± 0.39^a^	2.34 ± 0.34^a^	2.48 ± 0.41^a^	2.51 ± 0.12^a^	2.86 ± 0.41^a^
	8 weeks	1.70 ± 0.22^a^	1.75 ± 0.15^a^	1.03 ± 0.26^ab^	0.59 ± 0.33^b^	1.28 ± 0.19^ab^	1.73 ± 0.27^a^

^*^Means carrying different letters with the same raw are significantly different (*p* ≤ 0.05)

### Tissue oxidative stress and total antioxidant capacity level

**Fig. 13:**
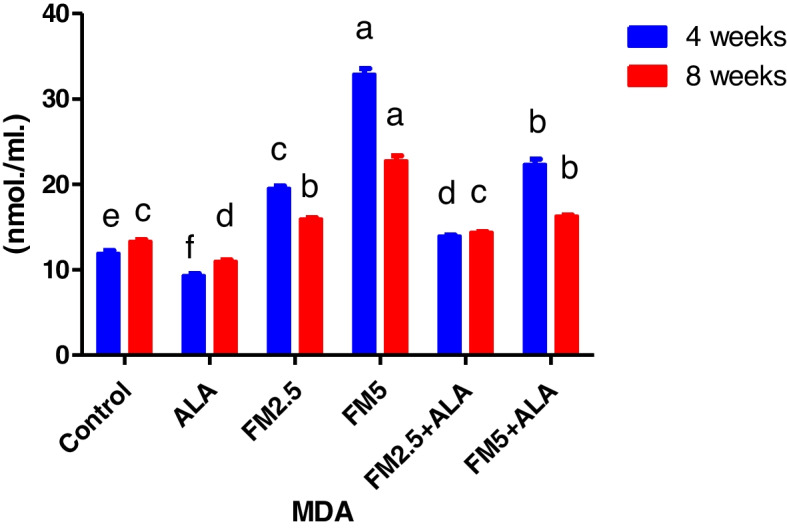
Effect of flunixin meglumine (FM) and alpha lipoic acid (ALA) on hepatic MDA levels on the 4 th and 8 th weeks of the experiment. There was a significant rise in the amount of MDA in the liver in the Flunixin meglumine-treated groups at doses of 2.5 and 5 mg/kg bwt compared to the control groups. The values are shown as the means ± S.E.M. (*n* = 6). In the FM- 5 + ALA and FM- 2.5 + ALA combination groups, the level was lower than in the treated groups alone

**Fig. 14:**
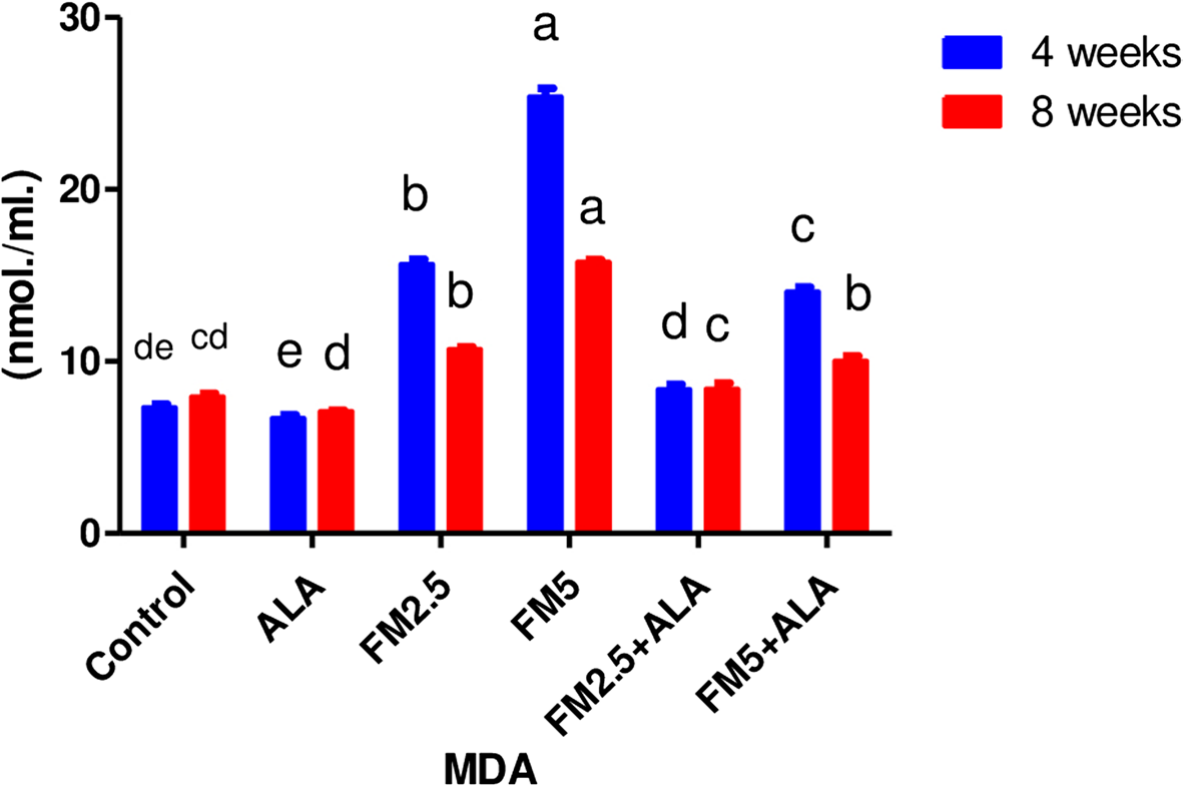
Effect of flunixin meglumine (FM) and alpha lipoic acid (ALA) on gastric MDA levels on the 4th and 8th weeks of the experiment. The results are shown as means ± S.E.M. (*n* = 6), and they show that the levels of MDA in the stomach were significantly higher in the groups that were given Flunixin meglumine at doses of 2.5 and 5 mg/kg bwt compared to the control groups. In FM-5 + ALA and FM-2.5 + ALA combination groups, the level was lower than in the treated groups alone

**Fig. 15:**
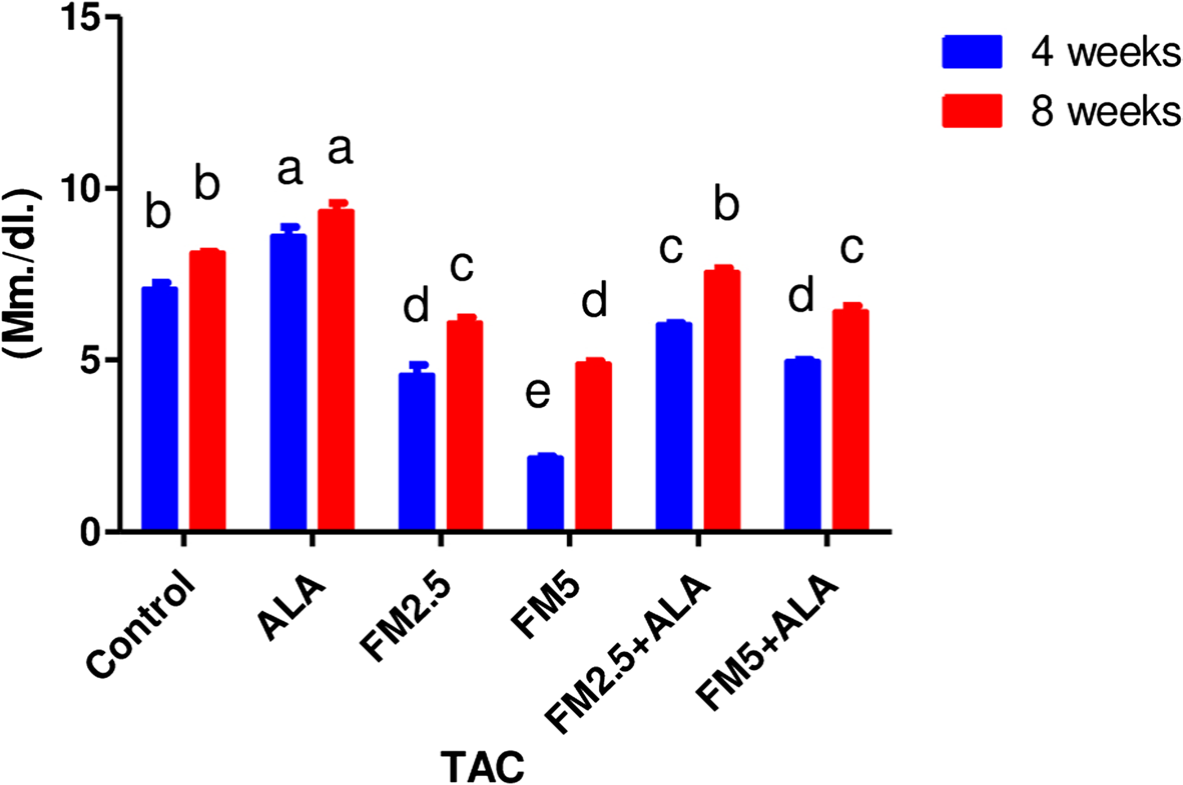
Effect of flunixin meglumine (FM) and alpha lipoic acid (ALA) on hepatic TAC levels at the 4th and 8th weeks of the experiment. The results show the means ± S.E.M. for a group of six rats. The hepatic TAC level was significantly lower in the Flunixin meglumine-treated groups when compared to the control groups when the dose was 2.5 and 5 mg/kg bwt. The levels were higher in FM-5 + ALA and FM-2.5 + ALA combination groups than in the treated groups alone

**Fig. 16:**
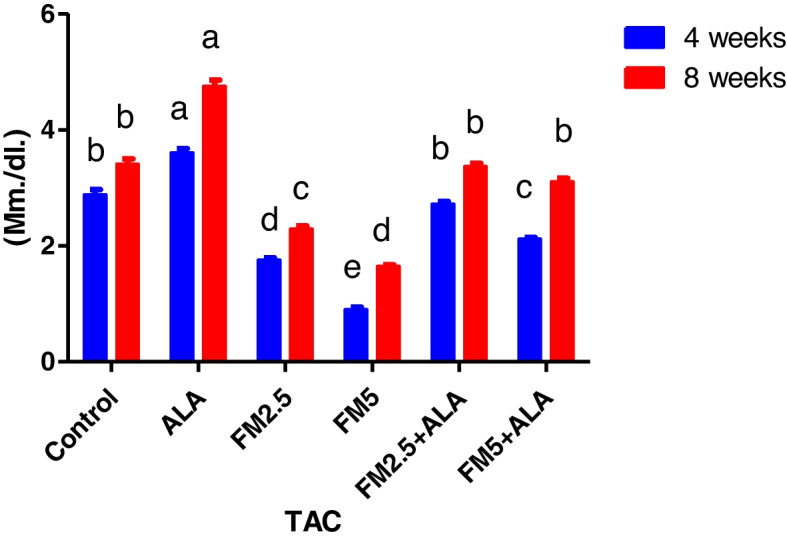
Effect of flunixin meglumine (FM) and alpha lipoic acid (ALA) on gastric TAC levels on the 4th and 8th weeks of the experiment. There was a significant drop in the amount of TAC in the stomach in the Flunixin meglumine-treated groups at doses of 2.5 and 5 mg/kg bwt compared to the control groups. The values are shown as the means ± S.E.M. (*n* = 6). The levels were higher in FM-5 + ALA and FM-2.5 + ALA combination groups than in the treated groups alone

Table 3 displays the therapeutic impact of FM and the ameliorative impact of ALA on oxidative stress in rat livers and stomachs. It was found that both doses of FM significantly (*p* ≤ 0.05) increased MDA content (Figs. 13 and 14) and decreased total antioxidant capacity (Figs. 15 and 16) compared to the control group. However, giving ALA along with FM completely protected the liver and stomach tissues from oxidative stress at the 4 th and 8 th weeks of the experiment. The results also showed that the liver and stomach tissues of rats that were given ALA had a significant (*n* ≤ 0.05) increase in TAC value and a significant (*p* ≤ 0.05) decrease in MDA content in the liver at the 4 th and 8 th weeks of the experiment compared to the control group.

**Table 3:** Effect of Flunixin meglumine (FM) and alpha lipoic acid (ALA) on hepatic and gastric MDA and TAC levels on 4^th^ and 8^th^ weeks of the experiment

Parameter	Period	Control	ALA	FM2.5	FM5	FM2.5 + ALA	FM5 + ALA
**Hepatic MDA and TAC levels**							
MDA (nmol./ml.)	4 Wk	11.91 ± 0.35^e^	9.31 ± 0.25^f^	19.52 ± 0.30^c^	32.89 ± 0.69^a^	13.94 ± 0.14^d^	22.33 ± 0.61^b^
	8 Wk	13.32 ± 0.18^c^	10.99 ± 0.16^d^	15.93 ± 0.15^b^	22.75 ± 0.59^a^	14.36 ± 0.09^c^	16.28 ± 0.12^b^
TAC (Mm./dl.)	4 Wk	7.07 ± 0.18^b^	8.61 ± 0.27^a^	4.56 ± 0.30^d^	2.15 ± 0.05^e^	6.03 ± 0.05^c^	4.96 ± 0.04^d^
	8 Wk	8.12 ± 0.04^b^	9.33 ± 0.25^a^	6.08 ± 0.16^c^	4.88 ± 0.09^d^	7.56 ± 0.12^b^	6.41 ± 0.17^c^
**Gastric MDA and TAC levels**							
MDA (nmol./ml.)	4 Wk	7.30 ± 0.21^de^	6.69 ± 0.19^e^	15.64 ± 0.29^b^	25.39 ± 0.50^a^	8.37 ± 0.30^d^	14.04 ± 0.28^c^
	8 Wk	7.95 ± 0.19^ cd^	7.10 ± 0.05^d^	10.69 ± 0.15^b^	15.76 ± 0.16^a^	8.40 ± 0.34^c^	10.02 ± 0.29^b^
TAC (Mm./dl.)	4 Wk	2.88 ± 0.09^b^	3.61 ± 0.07^a^	1.76 ± 0.04^d^	0.90 ± 0.05^e^	2.72 ± 0.05^b^	2.12 ± 0.03^c^
	8 Wk	3.41 ± 0.09^b^	4.75 ± 0.11^a^	2.29 ± 0.06^c^	1.65 ± 0.03^d^	3.37 ± 0.06^b^	3.11 ± 0.06^b^

^*^Means carrying different letters with the same raw are significantly different (*p* ≤ 0.05)

### Effect on gastric juice contents

**Fig. 17:**
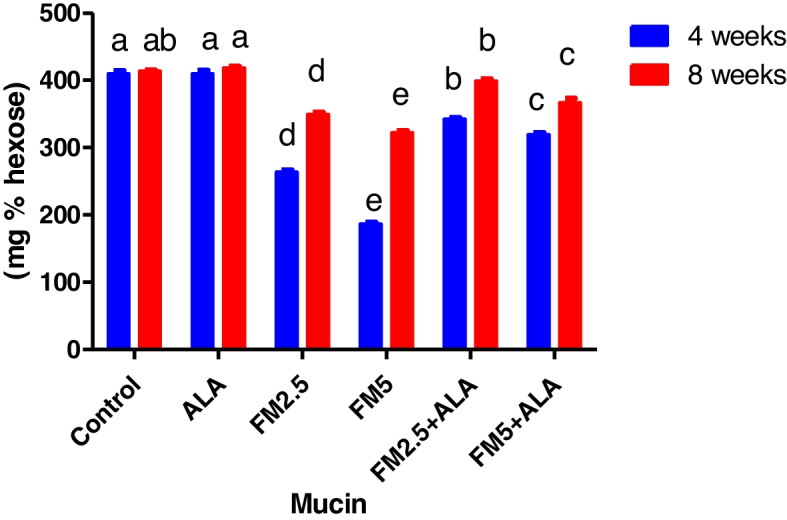
Illustrates the impact of flunixin meglumine (FM) and alpha lipoic acid (ALA) on the levels of gastric mucin during the 4th and 8th weeks of the experiment. The results are shown as mean ± S.E.M. for a group of six people. The groups that received Flunixin meglumine at doses of 2.5 and 5 mg/kg bwt showed significantly lower levels of gastric mucin compared to the control groups. The levels were higher in FM-5 + ALA and FM-2.5 + ALA combination groups than in the treated groups alone

**Fig. 18:**
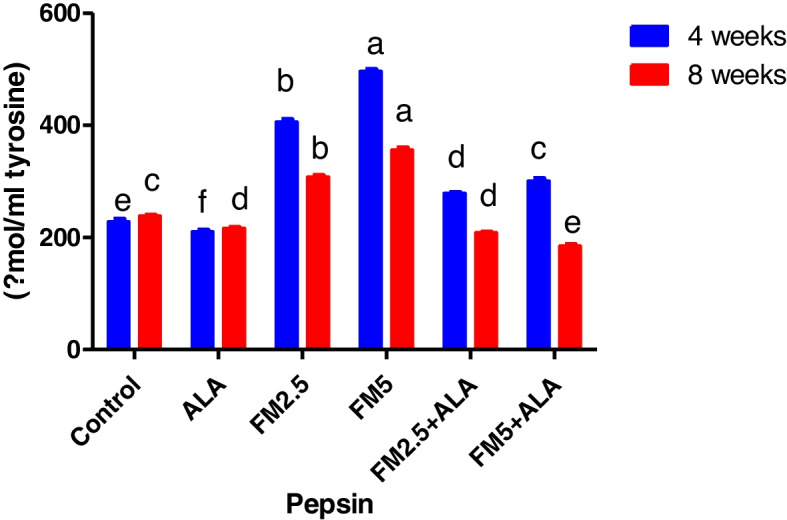
Effect of Flunixin meglumine (FM) and alpha lipoic acid (ALA) on gastric pepsin levels at the 4 th and 8 th weeks of the experiment. The results are shown as the means ± S.E.M. for a group of six rats. The levels of gastric pepsin were significantly higher in the groups that were given Flunixin meglumine at doses of 2.5 and 5 mg/kg bwt compared to the control groups. In FM- 5 + ALA and FM- 2.5 + ALA combination groups, the level was lower than in the treated groups alone

Table 4 showed that Flunixin Meglumine (FM) treatment at both 4 and 8 weeks led to significant decreases in gastric mucin levels (Fig. 17). We observed the highest decrease with FM at a dose of 5 mg/kg bwt, while the combination of FM- 5 with alpha-lipoic acid (ALA) showed a slightly lower decrease. FM treatment at a dose of 2.5 mg/kg bwt also resulted in decreased mucin levels, but to a lesser extent. ALA treatment alone did not significantly affect mucin levels compared to the control group. However, our study revealed that FM treatment at both 4 and 8 weeks caused significant increases in gastric pepsin levels (Fig. 18). We observed the highest increase with FM at a dose of 5 mg/kg bwt, while the combination of FM- 5 with ALA showed a slightly lower increase. FM treatment at a dose of 2.5 mg/kg bwt also led to increased pepsin levels, but to a lesser extent. In contrast, ALA treatment alone resulted in a slight decrease in pepsin levels compared to the control group.

**Table 4:** Effect of Flunixin meglumine (FM) and alpha lipoic acid (ALA) on gastric mucin and pepsin contents levels on 4^th^ and 8^th^ weeks of the experiment

Parameter	Period	Control	ALA	FM 2.5	FM 5	FM2.5 + ALA	FM5 + ALA
**Mucin (mg % hexose)**	4 wk	409.75 ± 4.92^a^	409.75 ± 5.61^a^	263.50 ± 3.19^d^	186.50 ± 2.80^e^	342.25 ± 2.46^b^	319.25 ± 3.02^c^
	8 wk	413.75 ± 1.96^ab^	418.50 ± 2.71^a^	349.50 ± 3.04^d^	322.25 ± 2.96^e^	398.75 ± 3.40^b^	366.75 ± 6.87^c^
**Pepsin (μmol/ml tyrosine)**	4 wk	227.75 ± 4.29^e^	210.25 ± 2.27^f^	405.75 ± 4.29^b^	496.25 ± 2.97^a^	278.50 ± 1.96^d^	300.75 ± 3.79^c^
	8 wk	238.00 ± 1.14^c^	216.00 ± 1.70^d^	307.75 ± 2.35^b^	355.50 ± 4.46^a^	208.00 ± 1.41^d^	184.0 ± 3.20^e^

^*^Means carrying different letters with the same raw are significantly different (*p* ≤ 0.05)

### Hepatic and gastric relative index weight

**Fig. 19:**
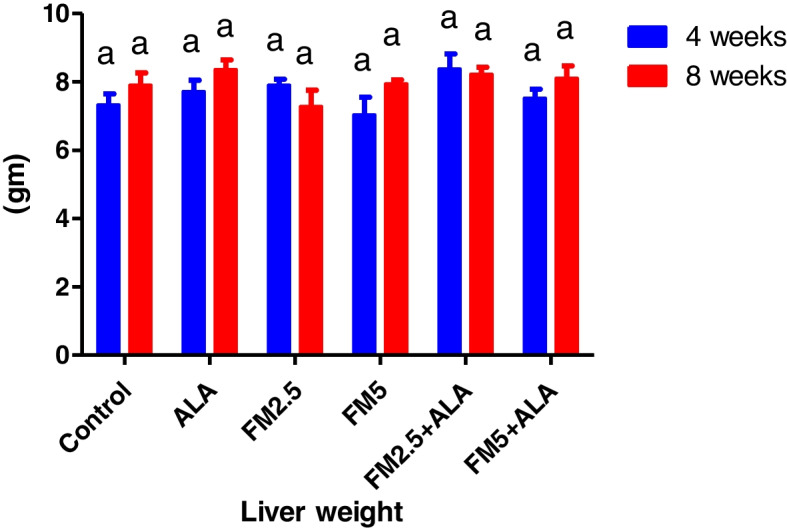
Illustrates the impact of flunixin meglumine (FM) and alpha lipoic acid (ALA) on liver weight during the 4 th and 8 th weeks of the experiment. Values are expressed as the means ± S.E.M. (*n* = 6), showing a non-significant change in the treated and control groups

**Fig. 20:**
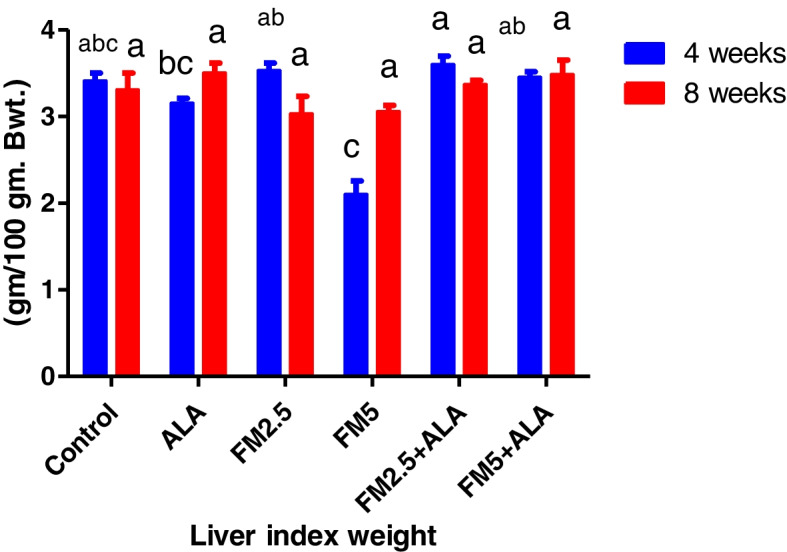
Effect of Flunixin meglumine (FM) and alpha lipoic acid (ALA) on liver index weight during the 4 th and 8 th weeks of the experiment. The results are shown as the means ± S.E.M. for a group of six animals. The liver index weight was significantly lower in the groups that were given Flunixin meglumine at doses of 2.5 and 5 mg/kg bwt at the end of the fourth week compared to the control groups. The levels were higher in FM- 5 + ALA and FM- 2.5 + ALA combination groups than in the treated groups alone

**Fig. 21:**
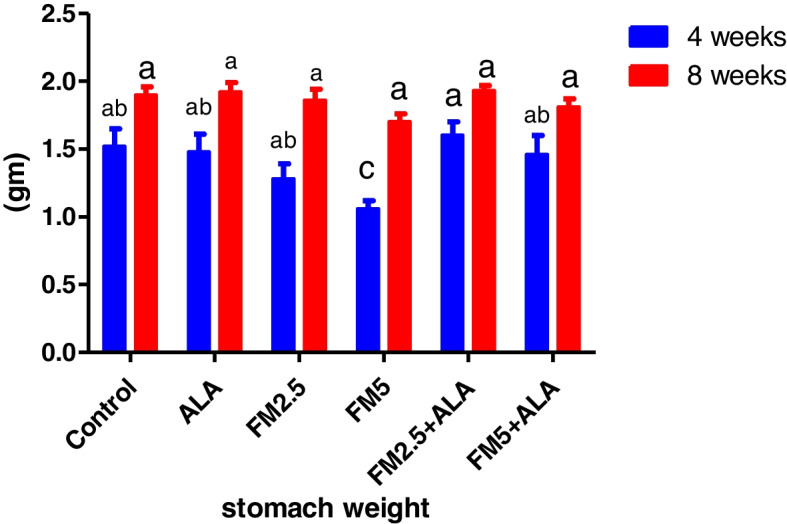
The effect of flunixin meglumine (FM) and alpha lipoic acid (ALA) on stomach weight in the 4 th and 8 th weeks of the experiment. The results are shown as the means ± S.E.M. for a group of six animals. The stomach weight was significantly lower in the groups that were given Flunixin meglumine at a dose level of 5 mg/kg bwt at the fourth week compared to the control groups. The level was higher in FM- 5 + ALA and FM- 2.5 + ALA combination groups than it was in the treated group alone

**Fig. 22:**
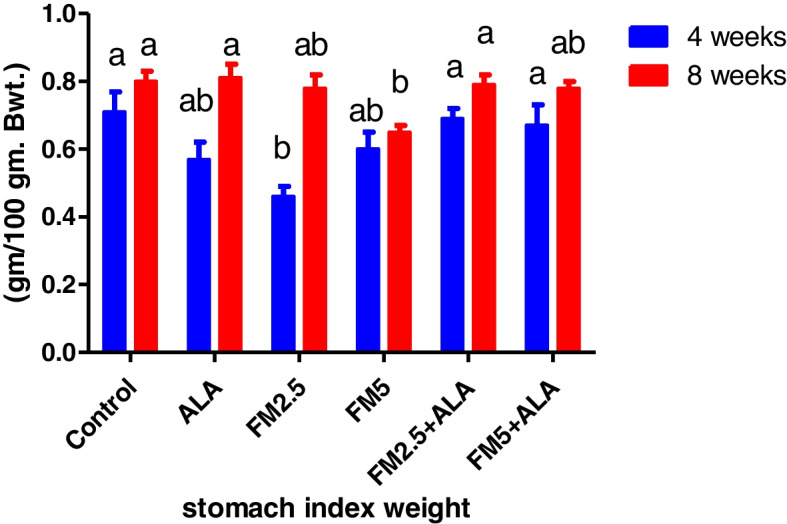
Effect of flunixin meglumine (FM) and alpha lipoic acid (ALA) on stomach index weight during the 4 th and 8 th weeks of the experiment. There was a significant drop in stomach index weight in the groups that were given Flunixin meglumine at a dose level of 2.5 mg/kg bwt at the 4 th week, and there was also a drop in the 8 th week in the groups that were given FM- 5 compared to the control groups. The level was higher in FM- 5 + ALA and FM- 2.5 + ALA combination groups than it was in the treated group alone

The administration of FM at the two-dose level did not result in any deaths or behavioral changes in the rat. There were significant (*p* ≤ 0.05) decreases in index weight of the liver of FM- 2.5 and FM- 5 at 4 th week and stomach in the FM- 2.5 at 4 th week and FM5 group at 8 th week versus control during the period of 4 and 8 weeks. The group treated with ALA along with FM2.5 and FM5 maintained this organ's relative index weight at the same levels as the control values. In addition, there was a decrease in stomach weight at the 4 th week in the FM- 5-treated group (Table 5) and (Figs. 19, 20, 21 and 22).

**Table 5:** Effect of Flunixin meglumine (FM) and alpha lipoic acid (ALA) on the hepatic and gastric weights and relative weights on 4^th^ and 8^th^ weeks of the experiment

Parameter	Period	Control	ALA	FM2.5	FM5	FM2.5 + ALA	FM5 + ALA
Liver weight	4 weeks	7.32 ± 0.33^a^	7.72 ± 0.33^a^	7.90 ± 0.18^a^	7.04 ± 0.51^a^	8.38 ± 0.44^a^	7.52 ± 0.27^a^
	8 weeks	7.90 ± 0.36^a^	8.36 ± 0.29^a^	7.28 ± 0.48^a^	7.94 ± 0.12^a^	8.22 ± 0.22^a^	8.10 ± 0.37^a^
stomach weight	4 weeks	1.52 ± 0.13^ab^	1.48 ± 0.13^ab^	1.28 ± 0.11^ab^	1.06 ± 0.06^c^	1.60 ± 0.10^a^	1.46 ± 0.14^ab^
	8 weeks	1.90 ± 0.058^a^	1.92 ± 0.07^a^	1.86 ± 0.08^a^	1.70 ± 0.058^a^	1.93 ± 0.04^a^	1.81 ± 0.06^a^
Liver index weight	4 weeks	3.41 ± 0.09abc	3.15 ± 0.06bc	3.53 ± 0.09ab	2.10 ± 0.16c	3.60 ± 0.10a	3.45 ± 0.07ab
	8 weeks	3.31 ± 0.19^a^	3.50 ± 0.12^a^	3.03 ± 0.20^a^	3.06 ± 0.07^a^	3.37 ± 0.05^a^	3.48 ± 0.17^a^
stomach index weight	4 weeks	0.71 ± 0.06^a^	0.57 ± 0.05^ab^	0.46 ± 0.03^b^	0.60 ± 0.05^ab^	0.69 ± 0.03^a^	0.67 ± 0.06^a^
	8 weeks	0.80 ± 0.03^a^	0.81 ± 0.04^a^	0.78 ± 0.04^ab^	0.65 ± 0.02^b^	0.79 ± 0.03^a^	0.78 ± 0.02^ab^

^*^Means carrying different letters with the same raw are significantly different (*p* ≤ 0.05)

### Gastric mucosal lesion

**Fig. 23:**
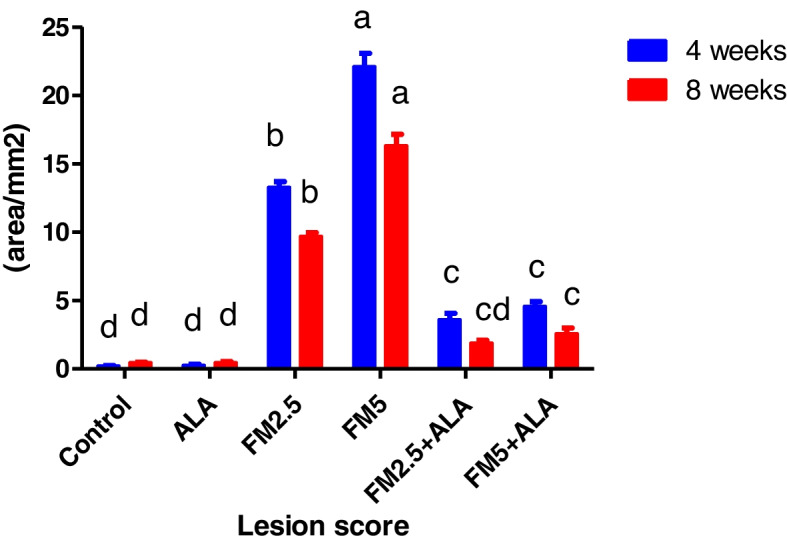
Effect of Flunixin meglumine (FM) and alpha lipoic acid (ALA) on gastric lesion score at 4 th and 8 th weeks of the experiment. The results are shown as the means ± S.E.M. for a group of six people. The groups that were given Flunixin meglumine at doses of 2.5 and 5 mg/kg bwt had a significantly higher gastric lesion score than the control groups. In the FM- 5 + ALA and FM- 2.5 + ALA combination groups, the level was lower than in the treated groups alone

**Fig. 24:**
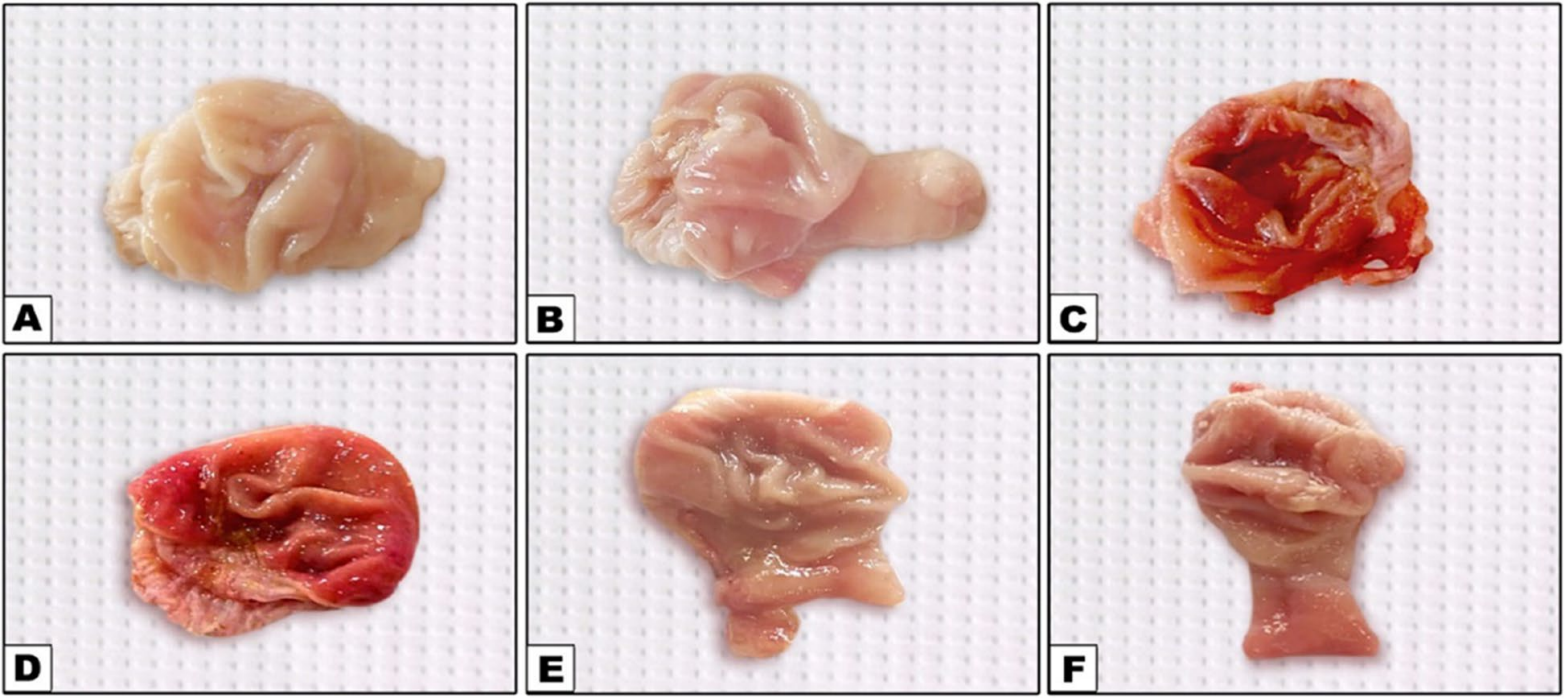
Representative macroscopic images of the gastric mucosa reveal the changes induced by Flunixin meglumine at two dose levels, showing a moderate degree of ulcer and severe congestion, as well as the protective action of alpha-lipoic acid on these gastric lesions: **A** Control, **B** Alpha lipoic acid, **C** FM- 2.5, **D** FM- 5, **E** FM- 2.5 + ALA, **F** FM- 2.5 + ALA

**Fig. 25:**
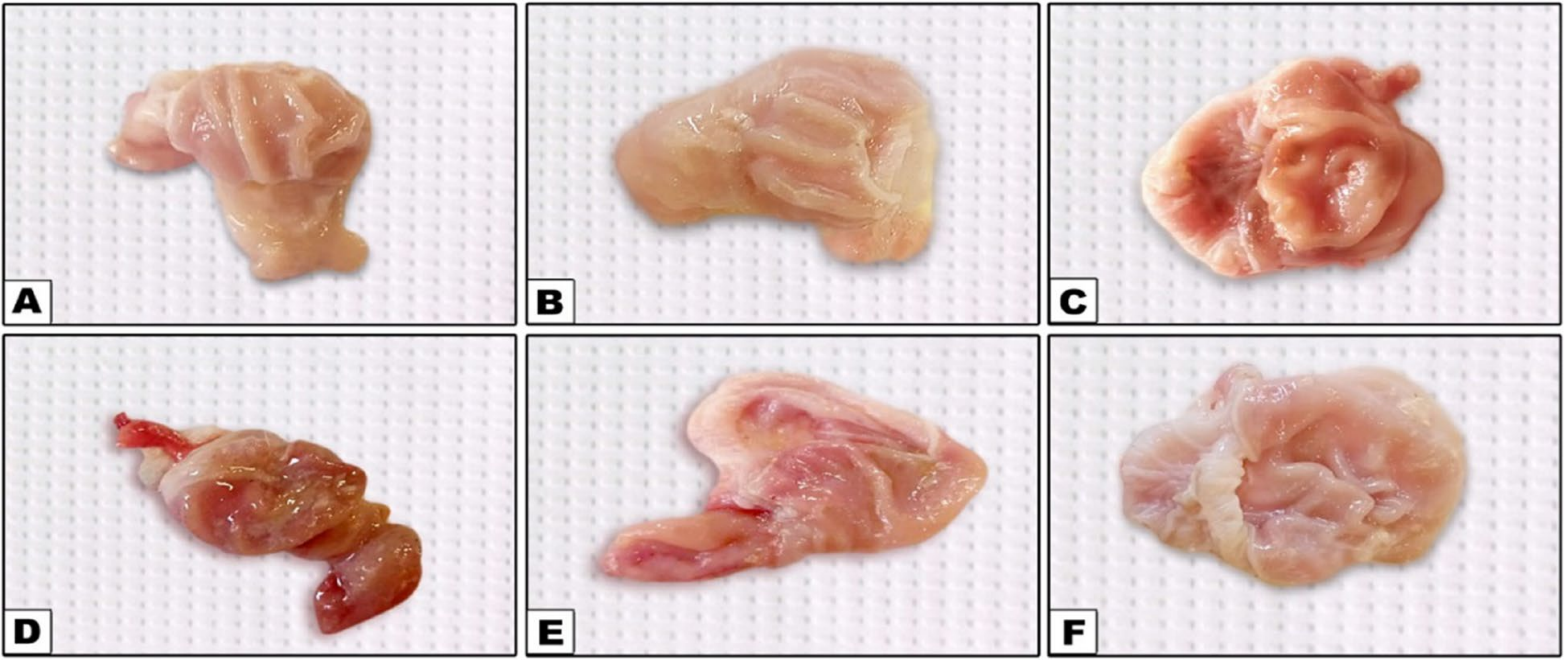
Representative macroscopic photos of the stomach mucosa demonstrate the alterations caused by flunixin meglumine at two different dosage levels. The photographs show congestion in the group treated with FM- 5 and the protective effect of alpha-lipoic acid on these gastric lesions. The experimental groups included: (**A**) Control, (**B**) Alpha lipoic acid, (**C**) FM- 2.5, (**D**) FM- 5, (**E**) FM- 2.5 + ALA, and (**F**) FM- 5 + ALA

Our study investigated the impact of flunixin meglumine (FM) and alpha-lipoic acid (ALA) on gastric mucosal lesions. The results revealed significant increases in gastric mucosal lesions at both 4 and 8 weeks in the groups treated with FM at a dose of 2.5 and 5 mg/kg bwt. The combination of FM- 5 with Alpha lipoic acid (ALA) showed a slightly lower elevation, while FM at a dose of 5 mg/kg bwt showed the highest increase in gastric mucosal lesions (Table 6) and (Figs. 23, 24 and 25).

**Table 6:** Effect of Flunixin meglumine (FM) and alpha lipoic acid (ALA) on gastric lesion scores on 4^th^ and 8^th^ weeks of the experiment

Parameter	Period	Control	ALA	FM 2.5	FM 5	FM2.5 + ALA	FM5 + ALA
Lesion score (area/mm^2)^	4wks	0.210 ± 0.05^d^	0.25 ± 0.09^d^	13.28 ± 0.44^b^	22.11 ± 0.99^a^	3.61 ± 0.47^c^	4.57 ±.036^c^
	8wks	0.44 ± 0.05^d^	0.46 ± 0.09^d^	9.70 ± 0.28^b^	16.32 ± 0.85^a^	1.88 ± 0.24^ cd^	2.57 ± 0.43^c^

^*^Means carrying different letters with the same raw are significantly different (*p* ≤ 0.05)

### Histopathological findings

**Fig. 26:**
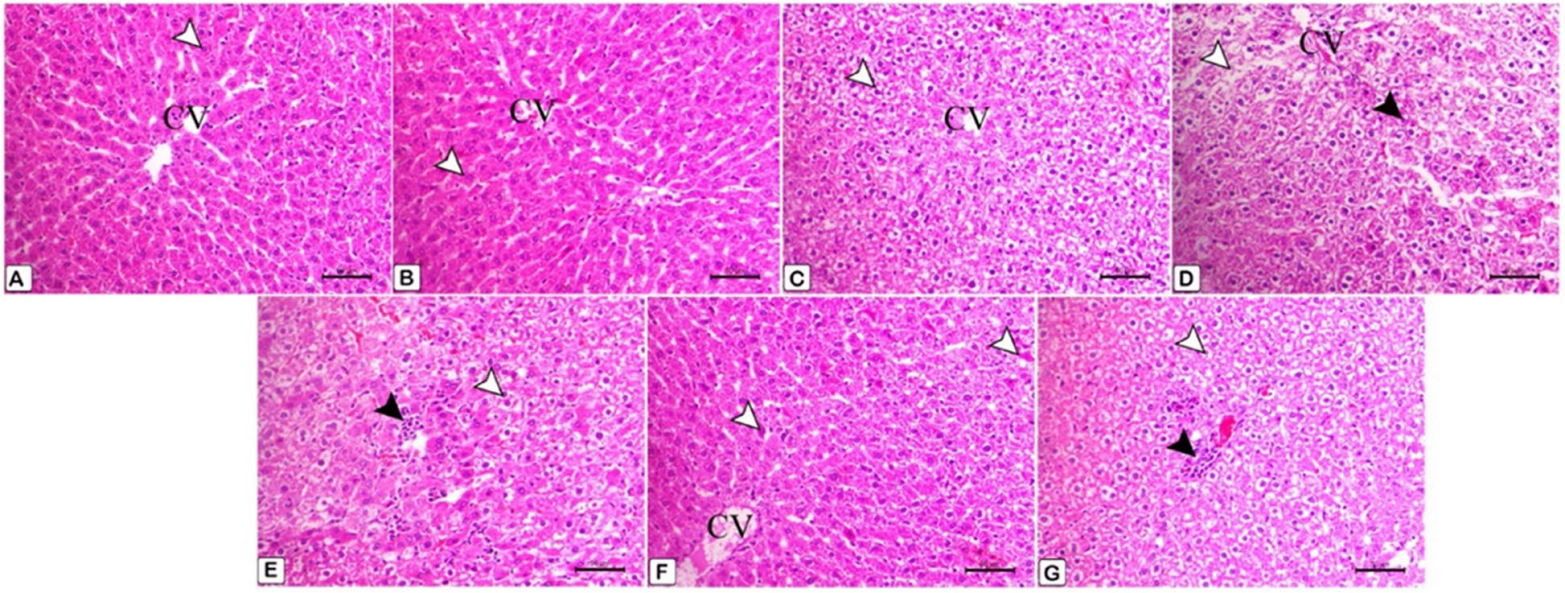
**A** The photomicrograph shows the normal histoarchitecture of the control group's liver tissue, with normal hepatocytes (arrowhead) organized in cords around the central veins (CV). Staining with H&E, scale bar = 50 μm. **B** Hepatocytes (arrowhead) in the normal histoarchitecture of the ALA group are grouped in cords around the central veins (CV) in this photomicrograph of liver tissue Staining with H&E, scale bar = 50 μm. **C** The photomicrograph shows hepatocytes with a modest degree of hydropic vacuolar alterations (arrowhead) and central veins (CV) in the liver tissue of the F group (2.5 mg/kg). Scale bar = 50 μm, H&E stain. **D** A photomicrograph of the liver tissue from the F group (5 mg/kg) reveals focal alterations in the vacuoles of the hepatocytes (white arrowhead) and nuclear pyknosis (black arrowhead), with central veins (CV) shown. Staining with H&E, with a scale bar of 50 μm. **E** A photomicrograph of the liver tissue from the F group (5 mg/kg) reveals intense hydropic vacuolation (white arrowhead) and focal necrosis (black arrowhead) in the liver, along with the infiltration of mononuclear cells, primarily macrophages. Scale bar = 50 μm, H&E stain. **F** Photograph of the liver tissue from the F (2.5 mg/kg) + ALA group (shown as CV) reveals normal hepatic cells with little apoptotic cells (arrowheads). Scale bar = 50 μm, H&E stain. **G** Liver tissue photomicrograph from the F (5 mg/kg) + ALA group reveals modest periportal infiltration of mononuclear cells (black arrowhead) and decreased hydropic vacuolar alterations of the hepatocytes (white arrowhead). Scale bar = 50 μm, H&E stain

Microscopic findings:

1. The liver:

At 4^th^ week: Figure 26 displays the changes on liver histoarchitecture from the various experimental groups.

**Fig. 27:**
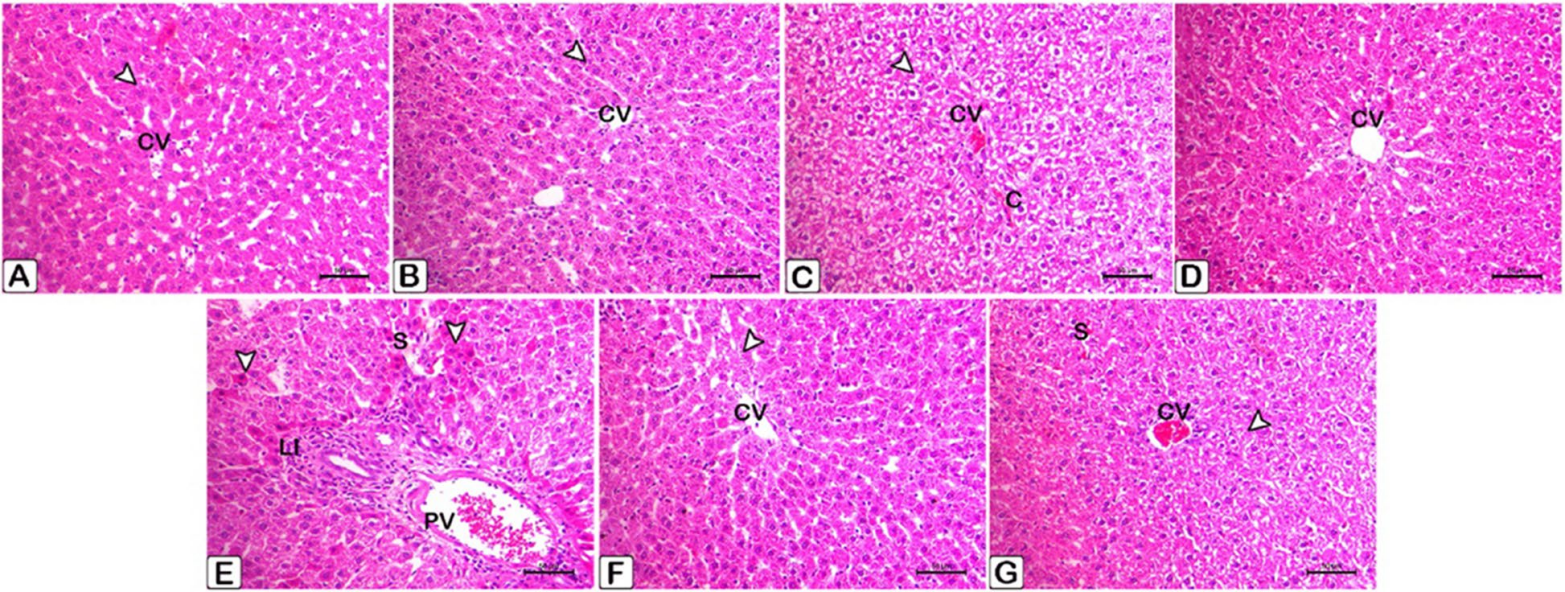
**A** The photomicrograph shows the normal histoarchitecture of the liver tissue from the control group. The normal hepatocytes (arrowhead) are arranged in cords around the central veins (CV)., H&E stain, scale bar = 50 μm. **B** Hepatocytes (arrowhead) in the normal histoarchitecture of the ALA group are grouped in cords around the central veins (CV) in this photomicrograph of liver tissue Staining with H&E, scale bar = 50 μm. **C** The photomicrograph shows hepatocytes with a modest degree of hydropic vacuolar alterations (arrowhead) and central veins (CV) in the liver tissue of the F group (2.5 mg/kg). Scale bar = 50 μm, H&E stain. Note the congestion (C) in the central vein and sinusoids. **D** A photomicrograph of the liver tissue from the F group (5 mg/kg) reveals. Staining with H&E, with a scale bar of 50 μm. **E** A photomicrograph of the liver tissue from the F group (5 mg/kg) reveals intense focal necrosis (white arrowhead) in the liver, along with periportal infiltration (LI) of mononuclear cells. Take note of the sinusoids getting wider and of both the sinusoids and the portal vein (PV) getting congested. Scale bar = 50 μm, H&E stain. **F** Liver tissue photomicrograph taken around the central vein (CV) in the F (2.5 mg/kg) + ALA group shows mostly normal hepatic cells with a small number of apoptotic cells (arrowheads).). Scale bar = 50 μm, H&E stain. **G** Liver tissue photomicrograph from the F (5 mg/kg) + ALA group decreased hydropic vacuolar alterations of the hepatocytes (white arrowhead), while congestion in the central vein (CV) and sinusoids (S) was still visible.. Scale bar = 50 μm, H&E stain

At 8 th week of the experiment: Fig. 27 displays the changes on liver histoarchitecture from the various experimental groups.

**Fig. 28:**
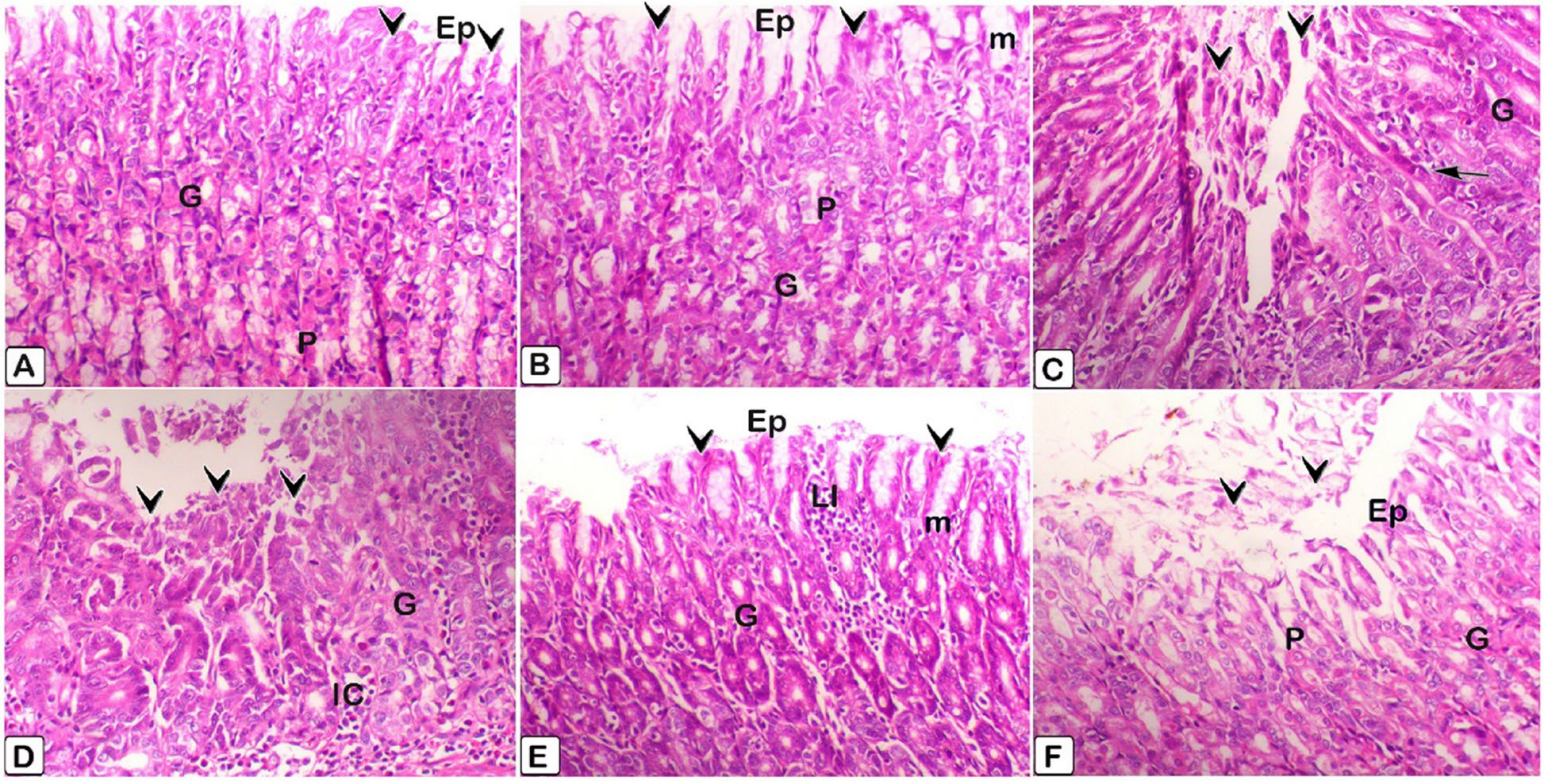
Histopathological sections of rat stomach tissue taken after the initial scarification. An H&E staining of the stomach tissue sections verified the alterations. **A** It was found that in rats that served as controls and **B** Gastric tissue in rats in the control group that received ALA doesn't show any abnormalities histologically, such as normal gastric glands (G), intact mucosa, no hemorrhages or epithelium exfoliations (Ep, arrowheads). **C** basal epithelial regeneration in the gastric gland (G, arrow), and ulceration and desquamation in mucous cells (arrowheads) are observed in the F group (2.5 mg/kg). **D** The stomach mucosal lining epithelium showed exfoliations (arrowheads) and substantial inflammatory cell infiltrations (IC) in the F (5 mg/kg) rat group. **E** The submucosal layer had decreased leucocyte infiltration (LI) and the epithelial surface remained intact in the F (2.5 mg/kg) + ALA group. **F** The F (5 mg/kg) + ALA group still showed blood vessel congestion (C), but there was less damage to the epithelial surface and less leucocyte infiltration (LI) of the submucosal layer than non-treated group. In both the control and treatment groups, you can see mucous neck cells (m) and parietal cells (P)

2. Stomach:

Figure 27 displays histopathological changes of rat stomach tissue, obtained after the initial scarification. Figure 28 displays histopathological stomach tissue changes of rats following the removal of therapies.

**Fig. 29:**
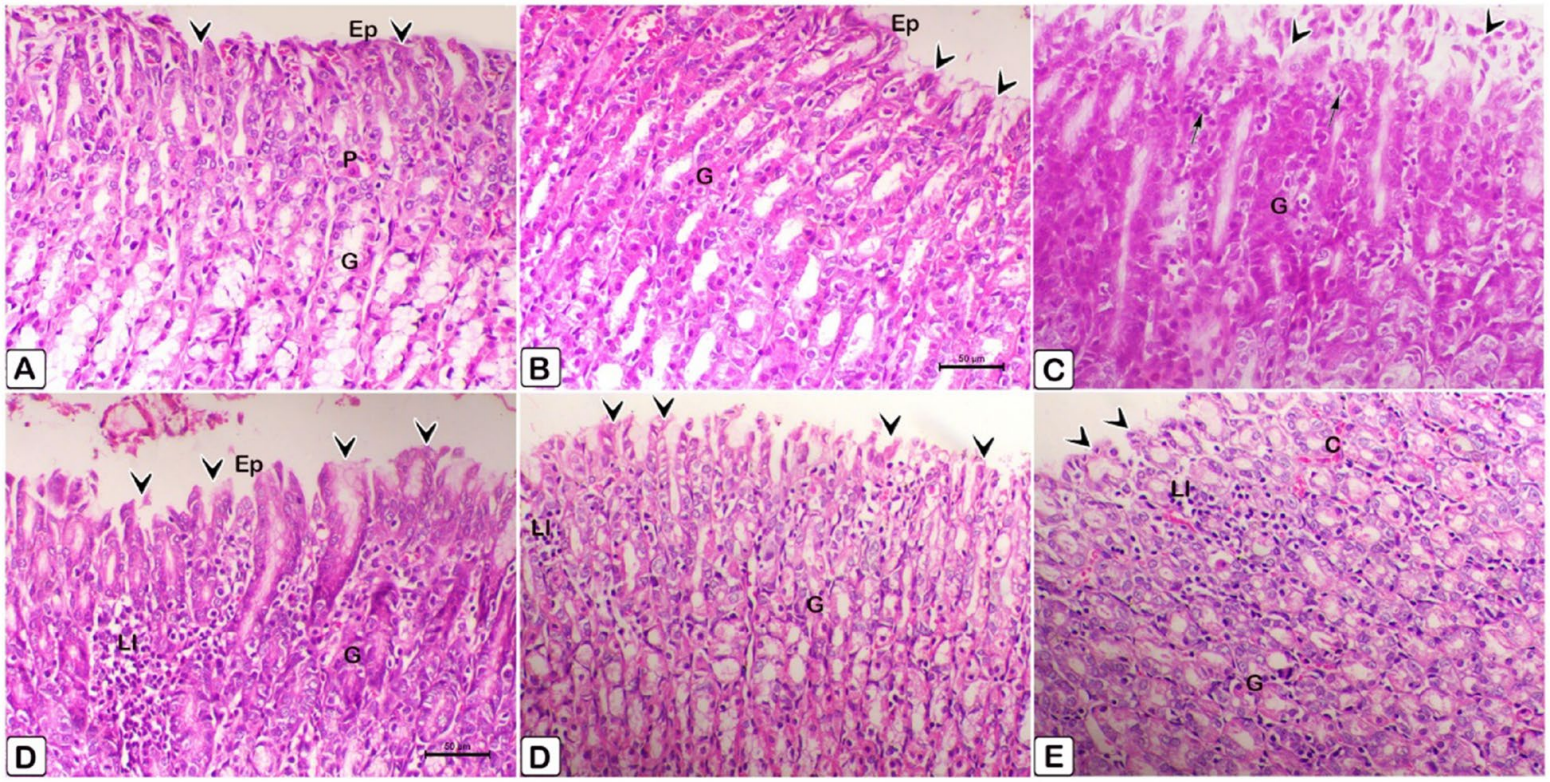
Histopathological stomach tissue sections of rats after withdrawal of treatments. The changes in the stomach tissue slide sections were confirmed by H&E staining. **A** In control rats and (**B**) In control produced ALA group rats, the gastric tissue is histologically normal, with intact mucosa, no hemorrhages or epithelium exfoliations (Ep, arrowheads), and normal gastric glands (G), note the presence of parietal cells (P). **C** In the F (2.5 mg/kg) group, there is ulceration and desquamation in mucous cells (arrowheads), as well as necrosis in the gastric gland (G) and a lymphocytes infiltration (arrows). **D** In the F (5 mg/kg) rat group, the lining epithelium of the gastric mucosa revealed massive exfoliations (arrowheads), as well as significant lymphocyte infiltration (LI). **E** In the F (2.5 mg/kg) + ALA group, there was reduce damage to the epithelial surface and there was less leucocyte infiltration (LI) of the submucosal layer. **F** The F (5 mg/kg) + ALA group showed less damage of the epithelial surface and reduced leucocyte infiltration (LI) of the submucosal layer while still demonstrating blood vessel congestion (**C**)

## Discussion

The point of this study is to investigate how flunixin meglumine therapy and its combination with alpha-lipoic acid can protect the liver and stomach in male rats used in experiments. Our study showed that giving Flunixin meglumine (FM) at doses of 2.5 and 5 mg/kg bwt over 14 days significantly increased the levels of NF-κB, TNF-α, and HMGB1 in the liver and stomach tissues after 4 and 8 weeks, compared to the control group. These findings suggest that long-term FM therapy activates the pro-inflammatory cytokine pathway in these tissues. The histological sections showed clear hydropic vacuolation and localized necrosis in the liver, along with the infiltration of mononuclear cells. The stomach tissues also show ulceration and desquamation in mucous cells (Figs. 27, 28 and 29). Some genes that are linked to human diseases, like inflammation, cancer, and autoimmune reactions, are controlled by the transcription factor NF-κB [48–50] NF-B regulates pro-inflammatory mediators like IL- 6 and COX- 2, mediating NF-inflammatory pathways [51]. The results corroborate previous findings that administering the therapeutic dosage of FM (2.5 mg/kg bwt) for 14 consecutive days resulted in increased levels of pro-inflammatory cytokines relative to the control group, although to a lesser extent than in the FM5 treatment group. The activation of these cytokines by FM may be somewhat reduced, even when administered at therapeutic doses. Bryant et al. [48] said that Flunixin meglumine (FM), a strong NSAID, lowers NF-kB activation, oxidative stress, and endotoxemia-related cytokine elevation. However, our results show the opposite. In contrast, the group treated with ALA in combination with FM during the 4 th and 8 th weeks of the trial exhibited reduced levels of pro-inflammatory cytokines in the stomach and liver compared to the groups treated with FM. This suggests that ALA may have a regulating effect on FM-induced cytokine release. Said et al. [52] reported that, administration of LA therapy reduces inflammation by blocking NF-κB, slows down the production of IL- 6 and COX- 2 proteins, and raisines IL- 10 levels in the testicles. Ying et al.'s [53] study shows that alpha-lipoic acid (ALA) stops human umbilical vein endothelial cells from activating NF-kB when TNFa is present. Crucially, ALA suppressed NF-kB activity regardless of its antioxidant properties. Alpha-lipoic acid (LA) activates signal transduction pathways and stimulates transcription factors, implying that LA internalization may occur through ligand-receptor interaction [54]. The results make a strong case for FM's ability to change the NF-κB, TNF-α, and HMG signaling pathways, all of which are very important for managing inflammatory reactions. Our results show that serum biochemical parameters related to liver function changed significantly in the groups that were given flunixin meglumine (FM) alone and FM combined with alpha-lipoic acid (ALA).We observed significant increases in the levels of alanine aminotransaminase (ALT), aspartate aminotransaminase (AST), and alkaline phosphatase (ALP) in groups that received FM at dosages of 2.5 and 5 mg/kg bwt for 14 consecutive days, respectively, compared to the control group. Alanine aminotransferase (ALT) and aspartate aminotransferase (AST) are enzymes predominantly found in hepatocytes. When their levels are high in the bloodstream, it suggests liver injury or dysfunction, as explained by Prabu et al. [55]Moreover, the increase in enzyme levels was more significant in group 4 (FM 5 mg/kg bwt) than in group 3 (FM 2.5 mg/kg bwt). These findings suggest that the increased FM dosage had a more pronounced effect on liver function and resulted in more substantial hepatotoxic consequences. The results of this study agree with those of Mohammed et al. [56], who found that giving rats flunixin by intraperitoneal injection at a dose of 2.5 mg/kg body weight significantly raised serum levels of ALT, AST, creatinine, and urea compared to the control group. The study findings revealed the detrimental effects of long-term flunixin use on the liver, as indicated by elevated levels of ALT, AST, ALP, and pathohistological alterations in both livers. Severe hydropic vacuolar changes of hepatocytes were seen, accompanied by pronounced nuclear pyknosisAdditionally, focal hepatic necrosis is accompanied by infiltration of mononuclear cells, predominantly macrophages. To examine the detrimental consequences of flunixin, it is necessary to understand the mechanism by which flunixin acts. Flunixin meglumine is a broad-spectrum inhibitor specifically targeting both cyclooxygenase- 1 (COX- 1) and COX- 2. Furthermore, it has received authorization for the management of several inflammatory and non-inflammatory disorders, such as arthritis, post-operative pain, and post-traumatic pain, in both human and animal subjects [8]. Cyclooxygenase enzymes help change arachidonic acid into prostanoids, which are chemicals like prostaglandins, prostacyclins, and thromboxane. Prostanoids play an important role in regulating several physiological processes in the gastrointestinal, circulatory, urogenital, and neurological systems, as well as immunity and inflammation [9]. In a big way, nonsteroidal anti-inflammatory drugs (NSAIDs), like flunixin, may cause two main types of liver damage: acute hepatitis, which includes symptoms like jaundice, nausea, and fever, as well as higher levels of serum transaminases; and chronic active hepatitis, which includes both serological and histopathological changes [57]. Moreover, groups 5 and 6, who received both FM and ALA, showed a decrease in ALT, AST, and ALP levels compared to those who only received FM treatment. The histopathological findings from male rats treated with Flunixin meglumine at both dosage levels, in conjunction with alpha lipoic acid, substantiate our results. There were predominantly normal liver cells with a limited number of apoptotic cells.

Alpha-lipoic acid, an antioxidant, has been shown to lessen the bad effects of fibrosis, a condition that can lead to cirrhosis and liver failure [58]. Morakinyo et al. [59] and Kaya-Dagistanli et al. [60] conducted studies that demonstrated ALA's strong defense against free radical-induced oxidative damage in the liver and kidney. The observed reduction in liver enzyme levels in the combination groups indicates that ALA may have diminished the hepatotoxic effects caused by fibromyalgia therapy. Furthermore, the FM-treated groups exhibited a notable decrease in the levels of total protein and globulin as compared to the control group after 8 weeks of the clinical trial. An observed reduction in these measures implies that the treatment with FM may have influenced protein production or metabolism. Furthermore, our findings aligned with the findings of Luna et al.'s [61] investigation. We observed no changes in the levels of total plasma protein, albumin, and globulin in a cohort of dogs administered flunixin meglumine at a dosage of 1 mg/kg for a duration of 3 days, measuring every 4 days. Likewise, there were no disparities noted among the groups at every designated time point. The liver is the principal organ responsible for the detoxification of several hazardous chemical compounds and medications, which contribute to oxidative stress. Elevated production of reactive oxygen species (ROS) makes mitochondrial membranes extremely vulnerable to oxidative damage. Moreover, reactive oxygen species (ROS) contribute significantly to the progression of endothelial damage and hepatic fibrosis [62]. In the 4 th and 8 th weeks of our study, rats that were given Flunixin meglumine (FM) at doses of 2.5 and 5 mg/kg body weight had significantly higher levels of Malondialdehyde (MDA) in their livers and stomachs, as well as significantly lower levels of antioxidant capacity overall. This was compared to the control group. Notably, male rats administered FM at a dosage of 5 mg/kg body weight showed the highest increase in MDA levels compared to the control group. In line with our findings, Yakan et al. [63] found that glutathione (GSH) and superoxide dismutase (SOD) levels dropped significantly in calves that were given Flunixin meglumine (2.2 mg/kg intravenously). Ellethy [64] also found that glutathione (GSH) levels dropped significantly in fish that were given ibuprofen and diclofenac. They also saw a drop in superoxide dismutase (SOD) and GSH levels in rats that were given celecoxib, a non-steroidal anti-inflammatory drug. The reported outcomes can be attributed to the potential neurotoxicity of the medication used in the investigation. Lipid peroxidation can significantly compromise the structural and functional integrity of cell membranes [65]. In our study, male rats that were given Alpha-lipoic acid alone had lower levels of malondialdehyde (MDA) in their livers and much higher levels of total antioxidant capacity in their livers and stomachs than the control group at both the 4 th and 8 th weeks of the oral administration of alpha-lipoic acid, combined with the subcutaneous administration of Flunixin meglumine at both dosage levels, resulted in a notable reduction in hepatic and gastric MDA levels and overall antioxidant capacity compared to the groups treated only with Flunixin meglumine. We acknowledge alpha-lipoic acid (ALA) as a naturally occurring antioxidant with the potential to inhibit the development of disorders associated with oxidative stress. Its antioxidant capacities enable it to remove reactive oxygen species (ROS) from the environment [66]. Furthermore, ALA possesses the capacity to restore intracellular glutathione (GSH), vitamin C, vitamin E, and other endogenous antioxidants [67]. Alpha Researchers have studied alpha-linolenic acid (ALA) as a potential treatment to mitigate the harmful effects of heavy metals [68] and have used it as a therapeutic or protective agent in several liver-associated disorders [69]. In dings indicated that long-term use of flunixin meglumine led to oxidative stress, driven by elevated levels of hepatic and gastric MDA and reduced levels of TAC. Concurrent use of ALA influenced these effects. Prevalence of stomach ulcers caused using nonsteroidal anti-inflammatory medicines (NSAIDs) continues to be a notable clinical issue [70, 71]. The stomach mucosa needs cyclooxygenase isoenzymes to keep making prostaglandins so that it can keep getting enough blood flow and encourage mucus production [72]. However, nonsteroidal anti-inflammatory drugs (NSAIDs) hinder cyclooxygenase activity, thereby diminishing the mucosa's inherent defense against harm from both internal and external sources [73]. A mucus gel layer shields the gastrointestinal tract, performing important functions such as hydrating, providing mechanical protection, eliminating pollutants, and facilitating the regeneration of protective molecules within the mucus [74]. Mucous neck cells in the stomach and goblet cells in the intestine produce and release mucin glycoprotein, the main constituent of the mucus layer [75]. When compared to the control group, the study showed that rats given Flunixin Meglumine (FM) at both dosage levels (2.5 and 5 mg/kg b.wt.) had lower levels of mucin and higher levels of pepsin at both the 4 th and 8 th weeks of the experiment. Specifically, male rats administered with FM at a dosage of 5 mg/kg body weight exhibited the most significant elevation in mucin levels and reduction in pepsin levels in comparison to the control group. The outcomes of our study are consistent with the results given by Santhosh et al. [76]. In Wistar male albino rats, administering ibuprofen orally at a dosage of 50 mg/kg twice a day at a 12-h interval resulted in a notable rise in the number of lesions in the gastric mucosa, an increase in the volume of gastric juice and acidity, and a reduction in the activity of pepsin. This study presents data that are in direct opposition to the conclusions provided by Marcinkiewicz et al. [77]. We observed that the administration of acetaminophen at a dosage level of 4000 mg/day resulted in a notable decrease in pepsin levels and no significant alteration in mucin levels among 30 asymptomatic volunteers. Marcinkiewicz, et al., [77] showed that the first mucin levels were 53% higher in people who had endoscopic mucosal changes after taking 660 mg/d of naproxen than in people who did not have the disease. Finally, the point of this study was to find out if alpha lipoic acid (ALA) could protect male rats'livers and stomachs from damage caused by flunixin meglumine (FM). Treatment with FM resulted in notable elevations in liver and stomach markers linked to injury, inflammation, and decreased stomach weight. Nevertheless, the simultaneous treatment of ALA and FM significantly enhanced these changes. ALA supplementation lowered elevated levels of markers and restored stomach weight, suggesting that it protects against liver and stomach problems caused by FM. These findings emphasize the therapeutic capacity of ALA in reducing disruptions caused by fibromyalgia. ALA's fundamental processes and clinical uses require further investigation.

## Conclusion

In conclusion, this study investigated the protective effects of alpha lipoic acid (ALA) against hepatic and gastric impairments caused by flunixin meglumine (FM) in male rats. FM administration led to significant increases in liver and stomach markers associated with damage, inflammation, and reduced stomach weight. However, co-administration of ALA with FM effectively improved these alterations. ALA supplementation attenuated elevated marker levels and restored stomach weight, indicating its protective role against FM-induced hepatic and gastric impairments. These findings highlight the therapeutic potential of ALA in mitigating FM-induced disturbances. Further research is needed to understand the underlying mechanisms and clinical applications of ALA.

## Data Availability

The datasets used and analyzed during the current study are available from the corresponding author upon reasonable request.

## Abbreviations

ALA: Alpha-lipoic acid
ALP: Alkaline phosphatase
ALT: Alanine aminotransferase
AST: Aspartate aminotransferase
COX: Cyclooxygenase
FM: Flunixin meglumine
Hb: Hemoglobin
HMGB1: High mobility group protein B1
NF-kb: Nuclear factor kappa-b
ROS: Reactive oxygen species
TNF-α: Total antioxidant capacity
TNF-α: Tumor necrosis factor-alpha
